# Uridine 5’-monophosphate (UMP) synthesis connects nucleotide metabolism to programmed cell death in *C. elegans*

**DOI:** 10.1038/s41418-025-01564-x

**Published:** 2025-09-03

**Authors:** Hang-Shiang Jiang, Hsiao-Fen Han, Cheng-Yi Chen, Kuan-Lun Hsu, Hung-Tsai Kan, Wan-Ying Lin, Mei-Hsuan Wu, Su-Yi Tsai, Jui-Ching Wu, Yi-Chun Wu

**Affiliations:** 1 Institute of Molecular and Cellular Biology, National Taiwan University, Taipei, Taiwan; 2 Department of Life Science, National Taiwan University, Taipei, Taiwan; 3 Research Center for Developmental Biology and Regenerative Medicine, National Taiwan University, Taipei, Taiwan; 4 Genome and Systems Biology Degree Program, National Taiwan University, Taipei, Taiwan; 5 Institute of Clinical Laboratory Sciences and Medical Biotechnology, College of Medicine, National Taiwan University, Taipei, Taiwan; 6 International Graduate Program of Molecular Science and Technology, National Taiwan University, Taipei, Taiwan; 7 Center for Computational and Systems Biology, National Taiwan University, Taipei, Taiwan; 8 Institute of Atomic and Molecular Sciences, Taipei, Taiwan; 9 Center for Advanced Computing and Imaging in Biomedicine, National Taiwan University, Taipei, Taiwan

**Keywords:** Development, Genetic interaction

## Abstract

Nucleotide metabolism is essential for fundamental cellular functions such as growth, repair and proliferation. Emerging evidence suggests that metabolic pathways also influence programmed cell death (PCD), though the underlying mechanisms remain poorly understood. One model organism that has provided key insights into the regulation of PCD is *Caenorhabditis elegans* (*C. elegans*). In this nematode, apoptosis is often initiated through asymmetric cell division (ACD), a process that unequally distributes fate determinants between daughter cells to produce a larger surviving cell and a smaller cell destined for apoptosis. Here, we demonstrate that the simultaneous disruption of PCD and ACD leads to aberrant cell survival and the formation of extra hypodermal cells. Through a genetic screen in the *grp-1* ACD mutant background, we identified *pyr-1* as a regulator of PCD. *pyr-1* encodes the *C. elegans* carbamoyl-phosphate synthetase/aspartate transcarbamoylase/dihydroorotase (CAD) enzyme which catalyzes the rate-limiting step of de novo pyrimidine biosynthesis, producing uridine 5’-monophosphate (UMP). UMP is a critical metabolite for the synthesis of nucleotides, lipids and carbohydrates. Genetic analysis of UMP metabolic pathways, combined with exogenous nucleoside supplementation, confirms that UMP availability is essential for PYR-1-mediated PCD. Loss of *grp-1* induces cellular stress by disrupting fate determinant partitioning during ACD, whereas *pyr-1* mutations cause metabolic stress through UMP depletion. While both mutations independently activate autophagy, they function redundantly to upregulate the mitochondrial chaperone *hsp-6*. Knockdown of autophagy-related genes and *hsp-6* reveals that these pathways serve as compensatory mechanisms to protect against cell death in the *pyr-1; grp-1* double mutants. Collectively, our findings establish a direct link between metabolism and cell death, demonstrating how UMP availability and proper ACD coordinate apoptotic regulation and developmental outcomes. This study highlights the intricate interplay between metabolic homeostasis and PCD, providing new insights into the metabolic control of cell fate decisions.

## Introduction

Programmed cell death (PCD), or apoptosis, is a fundamental biological process that eliminates unnecessary or damaged cells, ensuring proper organismal development and tissue homeostasis [1, 2]. During embryogenesis, the removal of transient or excess cells helps shape growing tissues and organs. Besides development, PCD plays a crucial role in maintaining immune homeostasis by preventing autoimmune disorders and regulating inflammatory responses [3]. While the genetic and molecular mechanisms underlying PCD have been extensively characterized, its relationship with cellular metabolism remains a relatively underexplored frontier.

The nematode *Caenorhabditis elegans* (*C. elegans*) has been instrumental in uncovering the genetic and cellular basis of PCD. Its simple anatomy, transparent body, and well-mapped cell lineage provide unique advantages for directly observing cell fate decisions [4–6]. PCD in *C. elegans* is often determined by asymmetric cell division (ACD), which produces a larger surviving cell and a smaller cell fated for apoptosis [4, 5, 7]. Genes such as *grp-1* (general receptor for phosphoinositides-1) play critical roles in this process, particularly within neuroblast lineages, generating apoptotic cells and neurons [8]. *grp-1* encodes the *C. elegans* homolog of cytohesin, an Arf guanine nucleotide exchange factor (GEF) involved in regulating membrane trafficking and actin cytoskeletal dynamics. Although its exact function remains to be explored, *grp-1* is proposed to function at the plasma membrane, likely at the cytokinetic furrow between dividing cells, to promote the asymmetry of the neuroblast division [8]. Loss of *grp-1* partially disrupts ACD, causing some apoptotic cells to adopt their sister cell fate, which results in the formation of excess neurons. Mutations in the core PCD genes such as *egl-1* (egg laying defective 1) or *ced-3* (cell death abnormality gene 3) significantly enhance the extra neuron phenotype [8], suggesting that ACD and PCD function synergistically to ensure proper cell fate determination and lineage progression. Despite this evidence in neuroblast lineages, it remains unclear whether this synergy between *grp-1* and PCD activation/execution genes also operates during the formation and differentiation of non-neuronal lineages or in other contexts beyond development.

Increasing evidence indicates that metabolic activity regulates apoptosis, suggesting that metabolic cues may directly or indirectly modulate apoptotic pathways [9]. For example, nucleotide availability and energy status indicators like ATP and NAD^+^ are known to influence cell death pathways. ATP drives caspase activation and apoptosome formation during the execution of apoptosis [10–12]. NAD^+^ plays a vital role in signaling pathways that regulate enzymes involved in cell survival and death [13–15]. For instance, DNA damage activates Poly (ADP-ribose) polymerase (PARP) for DNA repair, a process that consumes NAD^+^ and reduces energy reserves, thereby sensitizing cells to PCD. These findings underscore the importance of nucleotide metabolism in regulating the balance between cell survival and apoptosis. Further emphasizing this connection between metabolism and PCD, human CAD (carbamoyl-phosphate synthetase, aspartate transcarbamylase, and dihydroorotase), the enzyme catalyzing the rate-limiting step in the de novo pyrimidine synthesis pathway, has been shown to undergo caspase 3-mediated cleavage during apoptosis in response to apoptosis inducers staurosporine or doxorubicin [16]. However, it remains unclear whether this cleavage plays a functional role in the apoptotic process.

In *C. elegans*, the CAD homolog PYR-1 catalyzes the first three steps of de novo pyrimidine synthesis and is essential for uridine 5’-monophosphate (UMP) production, which is required for cell proliferation and differentiation [17, 18]. Similar to human CAD, *C. elegans* PYR-1 contains five functional domains – GLN, CPS-A, CPS-B, ATC, and DHO [19] – that sequentially facilitate pyrimidine biosynthesis. The GLN, CPS-A, and CPS-B domains together form the glutamine-dependent carbamoyl-phosphate synthetase (CPSase), where the GLN (glutamine amidotransferase) domain hydrolyzes glutamine to generate ammonia, which is transferred to the CPS (carbamoyl-phosphate synthetase) domain and incorporated into bicarbonate to form carbamoyl phosphate with the aid of ATP. The ATC (aspartate transcarbamoylase) domain catalyzes the reaction of carbamoyl phosphate with aspartate to form carbamoyl aspartate. The DHO domain, dihydroorotase, catalyzes the cyclization of carbamoyl aspartate to dihydroorotate. After this, other enzymes catalyze dihydroorotate to uridine monophosphate (UMP), completing UMP synthesis. DHOD catalyzes the oxidation of dihydroorotate to orotate, while UMPS, a bifunctional enzyme, performs two critical reactions: first, it converts orotate to orotidine 5’-monophosphate (OMP) via its orotate phosphoribosyltransferase (OPRT) domain, and then it converts OMP to UMP through its orotidine 5’-phosphate decarboxylase (OMPDC) domain [20]. The *dhod-1* and *umps-1* genes encode homologs of DHOD and UMPS, respectively, while the *R12E2.11* gene encodes only the OPRT domain.

Despite these advances, most studies on the metabolic regulation of PCD have been conducted in in vitro cultured cell lines, leaving significant gaps in our understanding of how metabolic pathways influence PCD during whole-organism development. To address these gaps, we set up our study to broaden our understanding of the physiological relevance of metabolism over apoptosis. Here, we demonstrate that *grp-1*, in conjunction with PCD activation and execution genes, regulates the cell death fate of hypodermal cell lineages. To further investigate these interactions, we performed a sensitized genetic screen on *grp-1* and identified *pyr-1*, the *C. elegans* CAD, as a critical factor cooperating with *grp-1* to ensure proper PCD in these lineages. Our findings reveal that UMP is the key metabolite required for PYR-1-mediated PCD. We also provide evidence that reduced UMP levels trigger autophagy and upregulate the mitochondrial chaperone *hsp-6*, particularly in the *grp-1* mutant background. Our data suggest that these pathways serve as protective mechanisms against cell death.

## Results

### *grp-1(gm350)* and specific PCD-related mutations synergistically cause extra hypodermal cell formation and a bulged tail phenotype

**Fig. 1:**
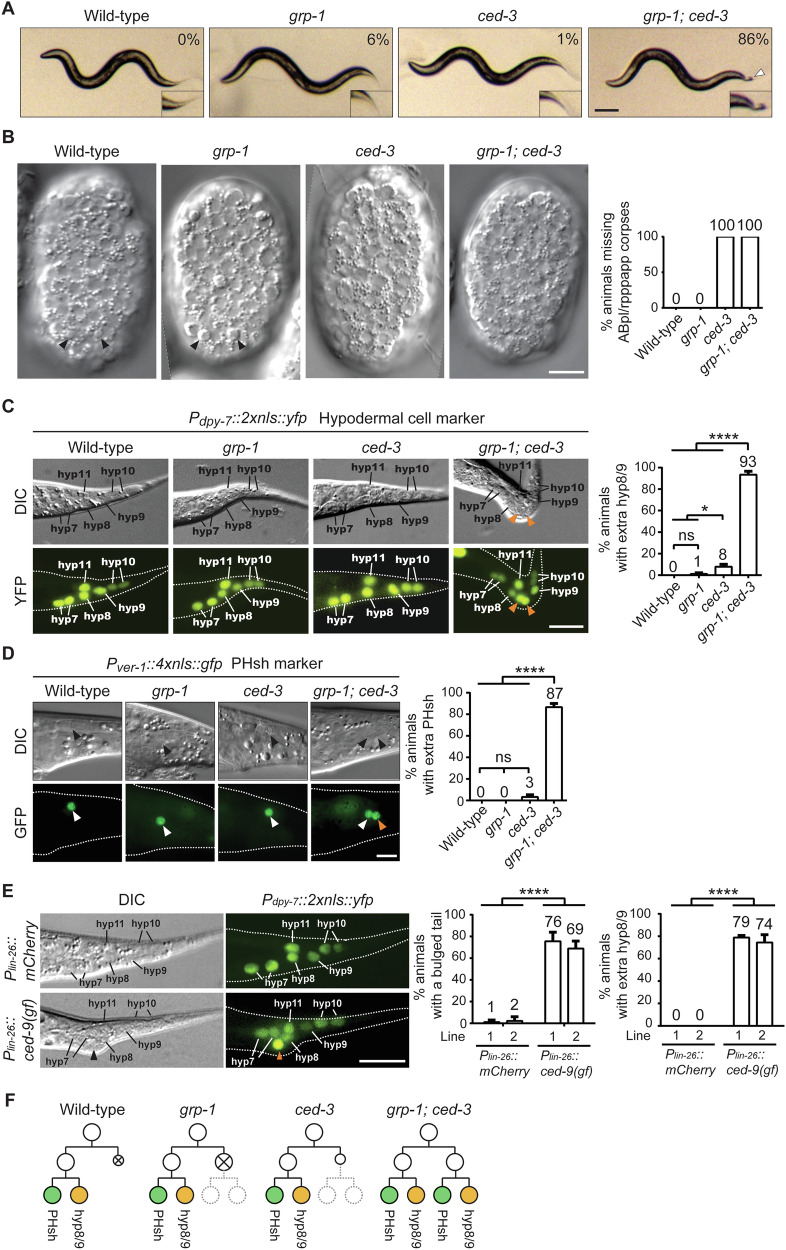
Extra hyp8/9 cells contribute to the bulged tail phenotype. **A** Dissecting microscope images of representative L4-stage nematodes in the wild-type and mutant backgrounds. The white arrowhead marks the bulged tail, which is shown at a 1.5-fold higher magnification in the inset. The percentages of animals exhibiting the bulged tail phenotype are shown in the upper right corner. Scale bar: 50 µm. **B** DIC images showing the presence or absence of the ABpl/rpppapp cell corpse in wild-type and mutant backgrounds. Black arrowheads indicate cell corpses. Scale bar: 10 µm. Percentage of animals lacking ABpl/rpppapp cell corpses is quantified and presented (*n* ≥ 16). DIC and fluorescence images of animals carrying the tail hypodermal cell marker *arIs99[P*_*dpy-7*_*::2xnls::yfp]* (**C**) or the PHsh marker *tpEx436[P*_*ver-1*_*::4xnls::gfp]* (**D**) in the indicated genetic background. Orange arrowheads indicate extra hyp8/9 (**C**) or PHsh (**D**). Scale bar: 10 µm. The percentages of animals displaying extra hyp8/9 (**C**) or PHsh (**D**) are shown as mean ± SD from three independent experiments (*n* = 30 per experiment). * indicates *P* < 0.05 and **** *P* < 0.0001 (one-way ANOVA with Tukey’s multiple comparisons test). ns indicates no statistical difference (*P* > 0.05). **E** DIC and fluorescence images of *grp-1; arIs99[P*_*dpy-7*_*::2xnls::yfp]* worms carrying the extrachromosomal array *P*_*lin-26*_*::mCherry* or *P*_*lin-26*_*::ced-9(gf)*. The percentages of animals exhibiting a bulged tail or extra hyp8/9 are shown as mean ± SD from three independent experiments for each line (*n* = 30 per experiment). **** indicates *P* < 0.0001 (one-way ANOVA with Tukey’s multiple comparisons test). Scale bar: 10 µm. **F** Schematic representation of the ABpl/rpppap cell lineage for the indicated genotypes. Cells fated to undergo PCD are marked with “x”, while differentiated functional cells are shown as color-filled circles. Dashed circles represent cells that occasionally escape PCD and subsequently differentiate. In wild-type animals, ABpl/rpppap divides asymmetrically to produce a larger anterior cell (ABpl/rpppapa) and a smaller posterior cell (ABpl/rpppapp). The anterior cell further divides and differentiates into PHsh and hyp8/9 cells, while the posterior cell undergoes PCD. In *grp-1* mutants, ABpl/rpppap divides symmetrically, producing two equal-sized cells. Despite this, the posterior cell typically undergoes PCD. However, in rare cases, the posterior cell survives and differentiates, leading to the generation of additional PHsh and hyp8/9 cells. In *ced-3* mutants, ABpl/rpppapp survives but does not further differentiate. In *grp-1; ced-3* mutants, ABpl/rpppapp survives and differentiates, resulting in a high penetrance of extra PHsh and hyp8/9 cells. Alleles used: *grp-1(gm350)* and *ced-3(n717)*.

The well-established function of *grp-1* in ACD of neuronal cells [8] led us to hypothesize if *grp-1* also played a role in PCD in other contexts. Although we did not observe any distinct phenotypes in the single *grp-1(gm350)* or *ced-3(n717)* mutants, the *grp-1*; *ced-3* double mutant worms displayed a distinctive bulged tail phenotype (Fig. 1A). This phenotype was evident as early as the newly hatched L1 stage, pointing to an embryonic developmental defect potentially linked to disrupted PCD and ACD during tail morphogenesis. Given that neuronal cells are typically smaller than other cell types, such as hypodermal cells, and their overproduction may not cause overt morphological abnormalities, we hypothesized that the bulged tail phenotype of *grp-1; ced-3* likely arises from the abnormal survival of non-neuronal cells, particularly hypodermal cells that are fated to die at the tail tip.

To test if the survival of hypodermal cells was causing the bulged tail phenotype in *grp-1; ced-3* double mutants, we traced the lineage of ABpl/rpppap cells, located symmetrically on the left (ABplpppap) and right (ABprpppap) side of the embryonic posterior end. In wild-type embryos, these cells divide asymmetrically to generate a larger daughter cell, ABpl/rpppapa, which subsequently divides to produce a ventral hypodermal cell hyp8/9 and a glia-like phasmid sheath cell PHsh. The smaller daughter cell, ABpl/rpppapp, undergoes PCD [4, 5, 21] (Fig. 1F). We observed that the apoptotic ABpl/rpppapp cells appear as button-like cell corpses approximately 270 min after the first cleavage in the wild-type and *grp-1* lines (Fig. 1B, indicated by arrowheads). Interesting, the apoptotic ABpl/rpppapp cells were larger in *grp-1(gm350)* than those in the wild-type embryos. These cells were retained in *ced-3* and *grp-1; ced-3* mutants as evidenced by the lack of ABpl/rpppapp apoptotic cell corpses (Fig. 1B).

We next examined the phenotypes of the hyp8/9 and PHsh cells at the L4 stage using the markers *arIs99[P*_*dpy-7*_*::2xnls::yfp]* and *tpEx436[P*_*ver-1*_*::4xnls::gfp*], respectively (Fig. 1C, D). The numbers of hyp8/9 and PHsh cells in L4 wild-type larvae were comparable to those in the *grp-1(gm350)* larvae. In the *ced-3(n717)* mutant, the number of hyp8/9 cells was significantly higher than that observed in wild-type animals (Fig. 1C) but the number of PHsh cells in the mutant was comparable to that in wild-type animals (Fig. 1D). In the *grp-1(gm350); ced-3(n717)* double mutant, ABpl/rpppapp cells not only survived (Fig. 1B) but also divided and differentiated, generating additional hyp8/9 and PHsh cells (Fig. 1C, D). These results indicate that the surviving ABpl/rpppapp cells adopt the fates of their sister cells, generating excess hyp8/9 and PHsh cells (Fig. 1F).

To examine how tissue organization might be affected by the different mutations and the exact contribution of hypodermal cells and PHsh cells to the bulged tail, we examined the localization pattern of the AJM-1::GFP marker which localizes to the adherens junction of hypodermal cells and outlines their apical border [22]. We observed that large additional hypodermal hyp8/9 cells, occupied the bulged tail region in the *grp-1(gm350); ced-3(n717)* double mutant (Supplementary Fig. S1). This phenotype was absent in the wild-type larvae and respective single mutants and suggests that the appearance of the bulged tail is due to the formation of the extra hyp8/9 cells, rather than the additional smaller and anteriorly positioned PHsh cells. We next wanted to determine whether blocking PCD in ABpl/rpppapp cells in the *grp-1(gm350)* single mutant would be sufficient to induce the bulged tail phenotype. To this end, we generated a construct containing the PCD regulator *ced-9(gf)* cDNA or *mCherry* under the control of the eE1.3 element of the *lin-26* promoter, which is active in ABpl/rpppap and its daughter cells [23]. The construct was microinjected into the *grp-1(gm350)* mutants carrying the *arIs99[P*_*dpy-7*_*::2xnls::yfp]* marker. As shown in Fig. 1E, overexpression of the cell death inhibitor *ced-9(gf)* [24, 25] led to the formation of extra hyp8/9 cells and the bulged tail phenotype, while the mCherry control did not induce these effects. Two independent lines were analyzed, confirming the reproducibility of this phenotype. These results demonstrate that the abnormal tail morphology observed in the *grp-1(gm350); ced-3(n717)* double mutant is due to ectopic survival and transformation of ABpl/rpppapp cells into additional hyp8/9 cells.

Consistently, double mutants of *grp-1(gm350)* paired with *ced-9(n1950, gain-of-function)*, *egl-1 (n1084n3082)*, or *ced-4(n1162)* mutations, led to a disruption of somatic cell death [24, 26–28] and bulged tail morphologies (Table 1). Notably, even the weaker *ced-3(n2427)* mutation or the mild *ced-3(n2923)* allele, which caused 1.2 and 0 extra cells in the pharynx [21], respectively, resulted in a bulged tail phenotype in 92% and 43% of worms in the *grp-1(gm350)* mutant background. Furthermore, the *ced-8(n1891)* mutation, which delayed PCD and phosphatidylserine externalization [29–31], also caused the bulged tail in the *grp-1* mutant (Table 1). These observations indicate that the bulged tail phenotype is highly sensitive to disruptions in the activation or execution of PCD, such that even minor reductions in the activity of the PCD machinery can produce abnormal tail morphology in the *grp-1* mutant.

**Table 1:** Execution and timing control defects in PCD lead to the bulged tail phenotype in the grp-1 background.

Genotype	% animals with a bulged tail
Wild-type	0%
*grp-1(gm350)*	6%
Defects in execution of cell death result in an aberrant bulged tail	
*egl-1(n1084n3082)*	2%
*grp-1(gm350); egl-1(n1084n3082)*	87%
*ced-9(n1950)*	5%
*grp-1(gm350) ced-9(n1950)*	94%
*ced-4(n1162)*	8%
*ced-4(n1162) grp-1(gm350)*	94%
*ced-3(n717)*	1%
*grp-1(gm350); ced-3(n717)*	86%
*ced-3(n2427)*	0%
*grp-1(gm350); ced-3(n2427)*	92%
*ced-3(n2923)*	1%
*grp-1(gm350); ced-3(n2923)*	43%
Defects in timing control of cell death result in an aberrant bulged tail	
*ced-8(n1891)*	0%
*grp-1(gm350); ced-8(n1891)*	80%
Defects in engulfment do not cause an aberrant bulged tail	
*ced-1(e1735)*	0%
*ced-1(e1735); grp-1(gm350)*	8%
*ced-12(bz187)*	1%
*ced-12(bz187); grp-1(gm350)*	8%
Defects in DNA degradation do not cause an aberrant bulged tail	
*cps-6(ok1718)*	0%
*cps-6(ok1718); grp-1(gm350)*	8%
*cnt-1(tm2313)*	1%
*cnt-1(tm2313)*; *grp-1(gm350)*	12%

The percentages of animals displaying a bulged tail phenotype were assessed at the L3-L4 stages using a dissecting microscope for the indicated genotypes (*n* = 300 from three independent experiments).

In contrast, mutations impairing the later stages in the apoptotic pathway, such as cell-corpse engulfment (*ced-1* and *ced-12*) and apoptotic DNA degradation (*cps-6* and *cnt-1*), did not significantly induce a bulged tail phenotype in the *grp-1* background (Table 1). This distinction underscores the critical importance of the activation and execution phases of PCD, which act synergistically with *grp-1* to ensure proper tail morphogenesis.

### *pyr-1* mutations, like PCD mutations, cause a bulged tail in the *grp-1(gm350)* mutant

**Fig. 2:**
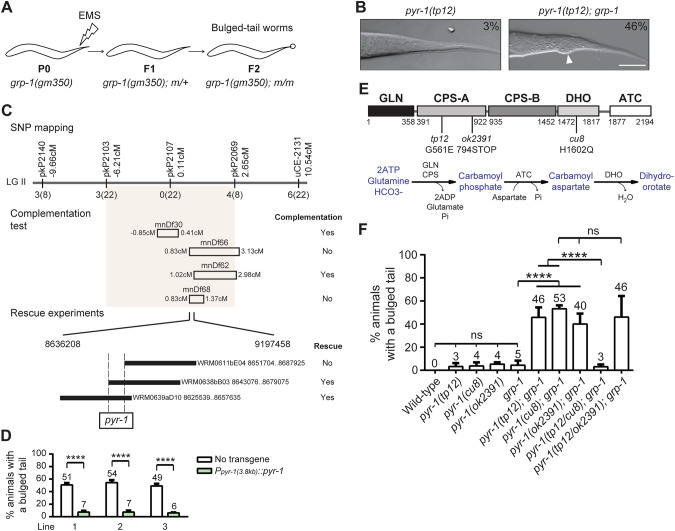
Mutations in *pyr-1* cause a bulged tail phenotype in the *grp-1(gm350)* mutant. **A** A schematic depicting the genetic screen designed to identify mutants with a bulged tail phenotype in the *grp-1(gm350)* sensitized background. One-day-old *grp-1(gm350)* adult worms were treated with ethylmethanesulfonate (EMS). The F1 progeny (heterozygotes) were isolated and their F2 progeny were screened for the bulged tail phenotype (homozygotes). **B** DIC images of *pry-1(tp12)* and *pyr-1(tp12); grp-1(gm350)* mutants. The percentages of mutants exhibiting the bulged tail phenotype are indicated on the top right corner. Scaled bar: 10 µm. **C** Mapping and cloning of *pyr-1*. The top panel shows a genetic map with five single nucleotide polymorphisms (SNPs) and the number of recombination events. The middle panel summarizes complementation tests using four deficiency strains. The bottom panel presents the results of rescue experiments using three fosmids (black rectangles) and a PCR-amplified *pyr-1* fragment (open rectangle). **D** The rescue results of the PCR-amplified *pyr-1* fragment are presented as the mean ± SD from three independent lines (*n* = 150 animals per line across three independent experiments). **** indicates *P* < 0.0001 (two-tailed *t* test). **E** Schematic representation of the PYR-1 protein domain structure. Amino acid positions and the mutations used in this study are indicated. Below, the catalytic steps of the de novo pyrimidine synthesis pathway mediated by different domains are shown. **F** Percentages of animals with a bulged tail phenotype for the indicated genotypes are shown. Data are presented as mean ± SD from at least three independent experiments (*n* = 50 animals per experiment). **** indicates *P* < 0.0001 (one-way ANOVA with Tukey’s multiple comparisons test). ns indicates no statistical difference (*P* > 0.05). The *grp-1* allele used is *gm350*.

To uncover key players that might work synergistically with GRP-1 in regulating PCD, we conducted a genetic screen on the *grp-1(gm350)* mutants and isolated worms with pronounced bulged tails, a marker for impaired PCD (Fig. 2A). From screening ~12,000 haploid genomes, we isolated 182 mutants that displayed a bulged tail phenotype with a penetrance exceeding 20%. Among these, four mutants were allelic to *egl-1*, four to *ced-4*, six to *ced-3*, and five to *ced-8*, demonstrating the robustness of this screen for isolating mutants defective in PCD. Of particular interest, we isolated the *tp12* allele, which caused a bulged tail phenotype in ~50% of *grp-1(gm350)* mutants (Fig. 2B). Using single nucleotide polymorphism (SNP) mapping, complementation testing, genomic DNA rescue, and whole genome sequencing experiments, we located *tp12* to the *pyr-1* locus (Fig. 2C and MATERIALS AND METHODS). A PCR product containing the *pyr-1* gene, including 3.8 kb upstream of the start codon and 2.1 kb downstream of the stop codon, was sufficient to rescue the bulged tail phenotype in *tp12; grp-1(gm350)* mutants (Fig. 2D).

Genome sequencing revealed that the *tp12* mutation results in a single amino acid substitution of a conserved glycine with glutamic acid (G561E) in the CPS-A domain (Fig. 2E). Additionally, two other *pyr-1* alleles, *cu8* and *ok2391*, previously identified as pharyngeal morphogenesis defective mutant [18] and isolated by the *C. elegans* Knockout Consortium, respectively, also caused bulged tails in the *grp-1(gm350)* background (Fig. 2F). The *ok2391* mutation produces a truncated PYR-1 protein containing the N-terminal GLN domain and an incomplete CPS-A domain, resulting in homozygous sterility (Fig. 2E). The *cu8* mutation results in a histidine-to-glutamine substitution (H1602Q) in the DHO domain [18] (Fig. 2E). Interestingly, *tp12* complemented *cu8* but not *ok2391* in the *grp-1(gm350)* background for the bulged tail phenotype (Fig. 2F), consistent with the molecular data that *cu8* and *tp12* affect distinct functional domains of CAD. These findings demonstrate that mutations in *pyr-1*, similar to mutations disrupting the activation or execution of PCD (Table 1), lead to the bulged tail phenotype in the *grp-1(gm350)* mutant.

### Mutations in *pyr-1* partially impair PCD

**Fig. 3:**
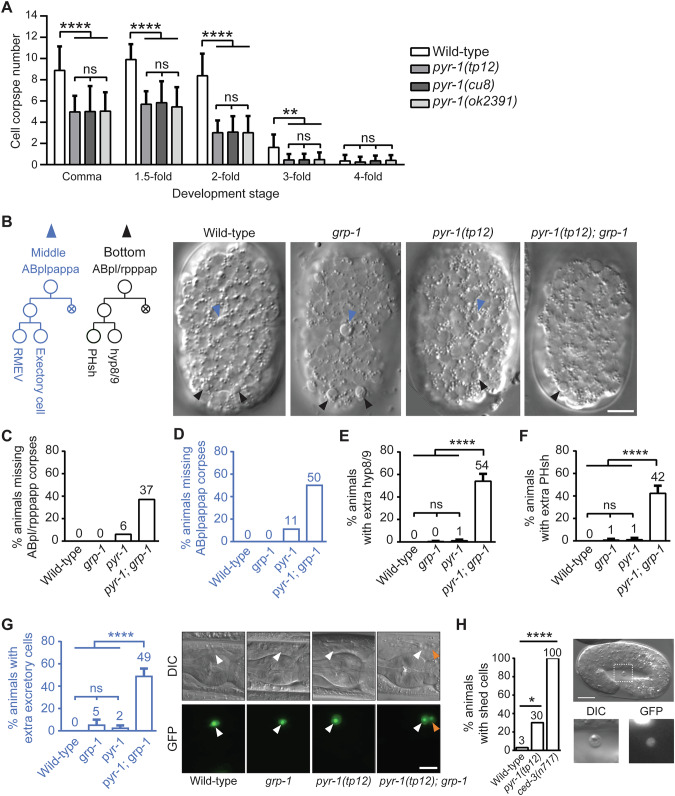
Loss of *pyr-1* disrupts PCD. **A** The number of cell corpses was scored at indicated developmental stages and is presented as mean ± SD (*n* ≥ 30). ** indicates *P* < 0.01 and **** *P* < 0.0001 (one-way ANOVA with Tukey’s multiple comparisons test). ns indicates no statistical difference (*P* > 0.05). **B** The ABplrpppap and ABplpappa lineages (left) and DIC images showing the presence or absence of ABpl/rpppapp and ABplpappap cell corpses in each genotype (right) are shown (*n* ≥ 16). Blue arrowheads point to ABplpappap cell corpses in the middle region of embryos, while black arrowheads mark ABpl/rpppapp cell corpses in the tail region. Scale bar: 10 µm. The percentages of embryos lacking ABpl/rpppapp (**C**) or ABplpappap (**D**) cell corpses are shown for each genotype (*n* ≥ 16). Quantification of animals with extra hyp8/9 carrying the marker *arIs99[Pdpy-7::2xnls::yfp]* (**E**) or extra PHsh carry the marker *tpEx436[Pver-1::4xnls::gfp]* (**F**) for each genotypes. Percentages are presented as mean ± SD from three independent experiments (*n* = 50 per experiment). **** indicates *P* < 0.0001 (one-way ANOVA with Tukey’s multiple comparisons test). ns indicates no statistical difference (*P* > 0.05). **G** DIC and fluorescence images of animals carrying the excretory cell marker *nIs434[P*_*pgp-12*_*::gfp]* in each genotype. White arrowheads indicate excretory cells, and the orange arrowhead highlights an extra excretory cell in the *pyr-1(tp12); grp-1(gm350)* mutant. Scale bar: 10 µm. Percentages of animals displaying extra excretory cells are presented as mean ± SD from three independent experiments (*n* = 50 per experiment). **** indicates *P* < 0.0001 (one-way ANOVA with Tukey’s multiple comparisons test). ns indicates no statistical difference (*P* > 0.05). **H** Percentages of three-fold stage embryos with shed cell (*n* ≥ 30). All strains contain the *egl-1* transcriptional reporter *bcIs37[P*_*egl-1*_*::his-24::gfp]*. * indicates statistically significant differences (*P* < 0.05) and ns denotes no significant difference (*P* > 0.05), determined by Fisher’s exact test. Scale bar: 10 µm. Allele used: *grp-1(gm350)*.

Having established that allelic mutants of *pyr-1* exhibit the bulged tail phenotype in the *grp-1(gm350)* mutant background, we next investigated if these *pyr-1* mutants also exhibit defects in PCD. Since most PCD events in *C. elegans* occur during embryogenesis [4, 5], we quantified apoptotic corpses at various embryonic stages in our mutants. All *pyr-1* allelic mutants show a significant reduction in the number of cell corpses starting at the comma stage to the three-fold embryonic stages, compared to wild-type embryos (Fig. 3A). This reduction in cell corpses does not appear to result from a delay in the execution of PCD, as significantly fewer corpses were observed in the *pyr-1* allelic mutants compared to wild-type embryos even at later embryonic stages, such as the three-fold stage.

To examine these PCD defects in more detail, we tracked the death of the ventrally located ABpl/rpppapp and ABplpappap cells in live embryos using four-dimensional differential interference contrast (DIC) microscopy (Fig. 3B). Compared to wild-type or *grp-1(gm350)* embryos which always displayed corpses for these cells, approximately 6% and 11% of *pyr-1(tp12)* mutant embryos lacked ABpl/rpppapp and ABplpappap corpses respectively (Fig. 3C, D). A more pronounced PCD defect was observed in the *pyr-1(tp12); grp-1(gm350)* mutants as 37% and 50% of these double mutant embryos lacked ABpl/rpppapp and ABplpappap corpses, respectively (Fig. 3C, D). We further examined the fates of these ABpl/rpppapp and ABplpappap cells at the L4 larval stage. As shown in Fig. 3E, F, the wild-type and single mutant animals showed little to no increase in the number of hyp8/9 and PHsh cells, as indicated by the markers *arIs99[P*_*dpy-7*_*::2xnls::yfp]* and *tpEx436[P*_*ver-1*_*::4xnls::gfp*] (Supplementary Fig. S2). In contrast, 54% and 42% of double mutants exhibited additional hyp8/9 and PHsh cells, respectively (Fig. 3E, F and Supplementary Fig. S2). These results suggest that, in *pyr-1(tp12); grp-1(gm350)* double mutants, the survival and differentiation of ABpl/rpppapp cells to extra hyp8/9 and PHsh cells is promoted. This mirrors the effects observed in mutations that disrupt the activation or execution of PCD (Table 1), albeit to a lesser extent.

Similarly, we examined whether the surviving ABplpappap cells of the double mutants would transform to its sister cell, generating an excretory cell at the L4 larval stage (Fig. 3B, left panel). Using the *nIs434[P*_*pgp-12*_*::gfp]* marker to identify excretory cells [32], we observed that 49% of *pyr-1(tp12); grp-1(gm350)* double mutants displayed an extra excretory cell, compared to only 2-5% in single mutants and 0% in wildtype (Fig. 3G).

To assess whether PCD defects extend to other lineages, we examined apoptosis in pharyngeal cell lineages and neuronal lineages. Among the cells fated to die, 16 cells in the anterior pharynx undergo PCD during mid-embryogenesis, such that virtually all wild-type animals lack extra surviving cells in this region by late larval stages [4, 5, 21] (Supplementary Fig. S3A). We found that *pyr-1(tp12)* mutants exhibited a slight increase in surviving pharyngeal cells relative to wild-type, with this phenotype significantly exacerbated in the *ced-3(n2427)* sensitized background [27, 33] (Supplementary Fig. S3A). We further analyzed apoptosis in embryonic neuronal lineages of Q.p, ABplapappp, and ABplapppap. In addition to apoptotic cells, the Q.p and ABplapappp lineages produce the touch neurons AVM/PVM and PLM, respectively, visualized using the *zdIs5[P*_*mec-4*_*::gfp]* marker [8, 34]; the ABplapppap lineage generates HSN and PHB neurons, visualized using *mgIs71[tph-1::gfp]* and *gmIs12[P*_*srb-6*_*::gfp]* markers, respectively [8, 35, 36] (Supplementary Fig. S3B–D). Consistent with the previous report, the *grp-1(gm350)* mutation led to extra AVM and PVM neurons [8]; however, the *pyr-1(tp12)* mutation did not enhance this phenotype (Supplementary Fig. S3B). Neither mutation affected the generation of extra PLM, HSN, or PHB neurons (Supplementary Fig. S3C, D). These results together indicate that apoptosis is affected in multiple, but specific, lineages in the *pyr-1(tp12); grp-1(gm350)* double mutants.

In addition to checking the fates of aberrant cells that survived developmental PCD in mutants, we also used the *P*_*egl-1*_*::gfp* marker to trace cells that escape death but are eventually shed from developing embryos [32]. Only 3% of wild-type embryos displayed shed cells as most cells destined for cell death undergo PCD. About 30% of *pyr-1(tp12)* embryos exhibited a cell-shedding phenotype compared to a whopping 100% of *ced-3(n717)* mutants which displayed shed cells (Fig. 3H). This observation, combined with the significant decrease in embryonic cell corpses (Fig. 3A) and the lineage-specific cell survival (Fig. 3B–G) in *pyr-1(tp12)* mutants indicates that *pyr-1* exerts moderate effects on PCD in individual embryonic lineages. These effects compound across multiple lineages and produce a more pronounced phenotype, as evidenced by the overall reduction in cell corpse numbers (Fig. 3A). Collectively, our data highlight the cumulative impact of *pyr-1* on PCD during embryogenesis.

### *pyr-1* acts through the de novo pyrimidine synthesis pathway to affect PCD

**Fig. 4:**
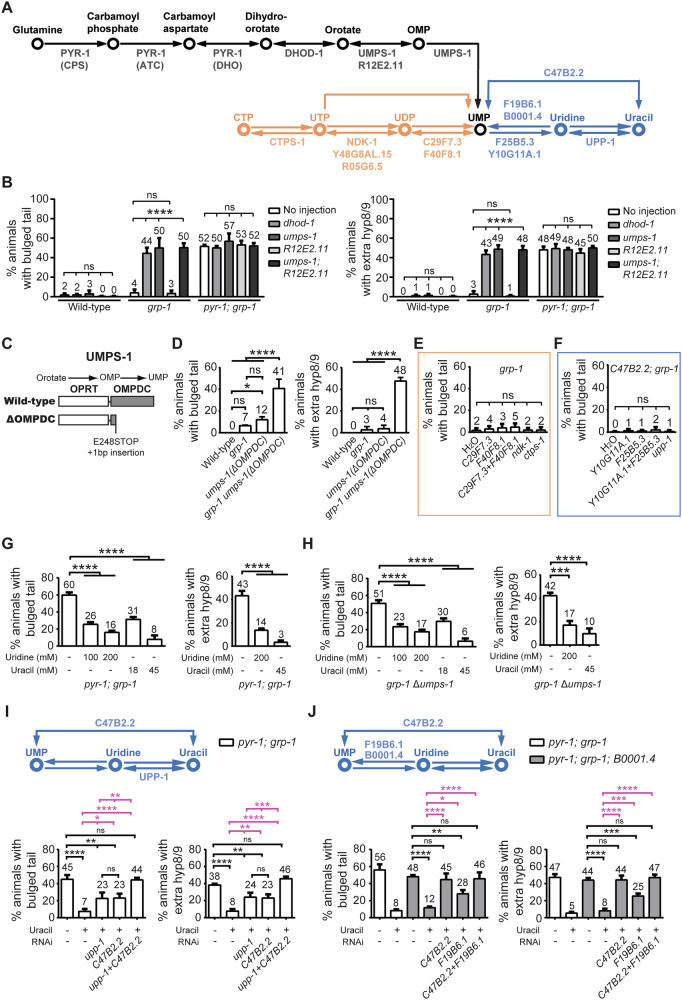
UMP is the key metabolite in PYR-1-mediated PCD. **A** Schematic of the de novo pyrimidine synthesis pathway (black) and the salvage pathway (blue), as adapted from WormFlux (https://wormflux.umassmed.edu/). These pathways converge at UMP, a central molecule in nucleotide metabolism. UMP can be further converted into derivatives such as UTP and CTP (orange), which serve as essential precursors for synthesizing sugars, RNA, and phospholipids. UTP and CTP can also be transformed into dUTP, dCTP, and dTTP, which are crucial for DNA synthesis. **B** Knockdown of the de novo pyrimidine synthesis pathway leads to a bulged tail phenotype in the *grp-1(gm350*) background. Animals of the indicated genotype were injected with dsRNA targeting *dhod-1*, *umps-1*, *R12E2.11* or *umps-1* plus *R12E2.11*. The percentages of animals displaying the bulged tail phenotype or extra hyp8/9 (*arIs99[P*_*dpy-7*_*::2xnls::yfp]*) are shown as mean ± SD (*n* = 50, from three independent experiments). **C**, **D** Schematic of the UMPS-1 protein with and without the OMPDC domain (**C**). CRISPR-mediated editing was performed in both wild-type and *grp-1(gm350)* worms to create genotypes harboring the truncated OMPDC domain. The percentages of animals displaying the bulged tail phenotype or extra hyp8/9 (*arIs99[P*_*dpy-7*_*::2xnls::yfp]*) for indicated genotypes are shown as mean ± SD (*n* = 30, from three independent experiments) (**D**). **E**, **F** In *grp-1(gm350)* mutants, animals were injected with dsRNA targeting *C29F7.3*, *F40F8.1*, *C29F7.3* plus *F40F8.1*, *ndk-1*, or *ctps-1* to inhibit the conversion of UMP to UDP, UTP, or CTP (orange highlighted region in Fig. 4A) (**E**). In *C47B2.2(tm2030); grp-1(gm350)* mutants, animal were injected with dsRNA targeting *Y10G11A.1*, *F25B5.3*, *Y10G11A.1* plus *F25B5.3*, or *upp-1* to block UMP conversion into uridine and uracil (blue highlighted region in Fig. 4A) (**F**). The percentages of animals with the bulged tail phenotype are shown as mean ± SD (*n* ≥ 30, from three independent experiments). **G**, **H** The effect of uridine or uracil supplementation at different concentrations was tested in *pyr-1(tp12); grp-1(gm350)* (**G**) and *grp-1(gm350) umps-1(*Δ*OMPDC)* (**H**) mutants. The percentages of animals displaying the bulged tail phenotype or extra hyp8/9 (*arIs99[P*_*dpy-7*_*::2xnls::yfp]*) are shown as mean ± SD (*n* ≥ 30, from at least three independent experiments). **I**, **J** In *pyr-1(tp12); grp-1(gm350)* mutants, dsRNA targeting *upp-1*, *C47B2.2*, or both was used to block uracil conversion to uridine or UMP (**I**). In *pyr-1(tp12); grp-1(gm350)* and *pyr-1(tp12); grp-1(gm350); B0001.4(tm2740)* mutants, dsRNA targeting *C47B2.2*, *F19B6.2*, or both was used to block uracil conversion to UMP (**J**). The percentages of animals with the bulged tail phenotype or extra hyp8/9 (*arIs99[P*_*dpy-7*_*::2xnls::yfp]*) are presented as mean ± SD (*n* ≥ 30, from at least three independent experiments). In all histograms, * indicates *P* < 0.05, **P < 0.01, ***P < 0.001, and ****P < 0.0001 (one-way ANOVA with Tukey’s multiple comparisons test). ns indicates no statistical difference (*P* > 0.05).

CAD, the human homolog of *pyr-1*, functions alongside enzymes dihydroorotate dehydrogenase (DHOD) and uridine monophosphate synthase (UMPS) to produce UMP in the de novo pyrimidine synthesis pathway (Fig. 4A). To investigate whether *pyr-1* regulates PCD in *C. elegans* through its role in the de novo pyrimidine synthesis pathway, we used RNA interference (RNAi) to knock down *dhod-1*, *umps-1*, and *R12E2*.11 enzymes of the UMP biosynthesis pathway and examined the effects on PCD impairments. Knockdown of *dhod-1* or *umps-1* and a combination of the two, but not *R12E2.11*, led to a bulged tail phenotype and extra hyp8/9 cells in the *grp-1(gm350)* background (Fig. 4B). Specifically, 44% and 50% of worms with *dhod-1* and *umps-1* knockdown, respectively, displayed these phenotypes, compared to less than 3–4% in control or *R12E2.11* knockdown worms. However, knocking down *dhod-1* or *umps-1* in *pyr-1(tp12); grp-1(gm350)* double mutants did not exacerbate these phenotypes (Fig. 4B). These results indicate that *dhod-1* and *umps-1* influence PCD through the same pathway as *pyr-1*, and their disruption likely impairs PCD through defective pyrimidine synthesis.

### The metabolite UMP is critical for PYR-1-mediated cell death

To further investigate the importance of the UMP biosynthesis pathway in for regulating PCD, we turned our attention to the orotate-OMP-UMP axis. The UMPS-1 enzyme, which converts orotic acid to UMP, has two domains of which the OMPDC domain catalyzes the final step of converting OMP to UMP in the de novo UMP synthesis (Fig. 4C) [20]. To assess the importance of the OMPDC domain in UMPS-1’s function in PCD, we used CRISPR-Cas9 to introduce a premature stop codon at the 248th codon of the *umps-1* gene. This edit, paired with a 1-bp insertion following the stop codon, generated a truncated UMPS-1 protein that lacked a functional OMPDC domain but had an intact OPRT domain (Fig. 4C). The resulting *umps-1 (*Δ*OMPDC)* mutant exhibited bulged tails in 41% of worms and extra hyp8/9 in 48% of worms in the *grp-1(gm350)* background (Fig. 4D). These results suggest that reduced UMP levels contribute to the abnormal survival of otherwise doomed cells in the *umps-1(*Δ*OMPDC); grp-1* double mutants.

UMP is sequentially converted to CTP through several enzymatic steps: C29F7.3 and F40F8.1 convert UMP to UDP, NDK-1, along with Y48G8AL15 and R05G6.5, converts UDP to UTP, and CTPS-1 converts UTP to CTP (Fig. 4A, orange). UMP can also be converted to uridine by F25B5.3 and Y10G11A.1, followed by a conversion to uracil by UPP-1, or directly from UMP to uracil by C47B2.2 (Fig. 4A, blue). We generated RNAi knockdown of enzymes involved in converting UMP to downstream metabolites such as CTP and uracil. Knockdown of *C29F7.3*, *F40F8.1*, *ndk-1*, or *ctps-1*, either individually or in combination, in the *grp-1(gm350)* mutant background did not result in bulged tail defects (Fig. 4E). Additionally, RNAi knockdown of F25B5.3 and Y10G11A.1, individually or in combination, in *C47B2.2(tm2030); grp-1(gm350)* double mutants also did not produce any bulged tail phenotypes (Fig. 4F). These findings indicate that neither the downstream metabolites of UMP nor its conversion pathways to CTP or uracil play a role in the bulged tail phenotype, further supporting the critical role of UMP itself in PYR-1-mediated PCD.

To further confirm that UMP depletion directly resulted in the bulged tail phenotype, we tested whether supplementation with exogenous uracil or uridine could rescue this phenotype. Indeed, the addition of exogenous uracil or uridine significantly reduced both the bulged tail phenotype and the presence of extra hyp8/9 cells in a dose-dependent manner in both *pyr-1(tp12); grp-1(gm350)* and *grp-1(gm350) umps-1(*Δ*OMPDC)* double mutants (Fig. 4G, H). In the salvage pathway, uracil can be converted stepwise to uridine via UPP-1 and subsequently to UMP via F19B6.1 and B0001.4, or directly converted to UMP via C47B2.2 (Fig. 4A) [20, 37]. Notably, RNAi knockdown of *upp-1* and *C47B2.2*, which blocks the conversion of uracil to uridine or UMP, completely abolished the rescue effect of uracil supplementation in *pyr-1(tp12); grp-1(gm350)* mutants (Fig. 4I). Furthermore, in *pyr-1(tp12); grp-1(gm350); B0001.4(tm2740)* triple mutants, RNAi knockdown of either *C47B2.2* alone or both *C47B2.2* and *F19B6.1*, which disrupt the conversion of uracil and uridine to UMP, also prevented uracil from rescuing the bulged tail phenotype and extra hyp8/9 cells (Fig. 4J). Collectively, these results demonstrate the importance of UMP in mediating *pyr-1*-dependent PCD.

### Disruption of both *pyr-1* and *grp-1* activates autophagy to prevent cell death

**Fig. 5:**
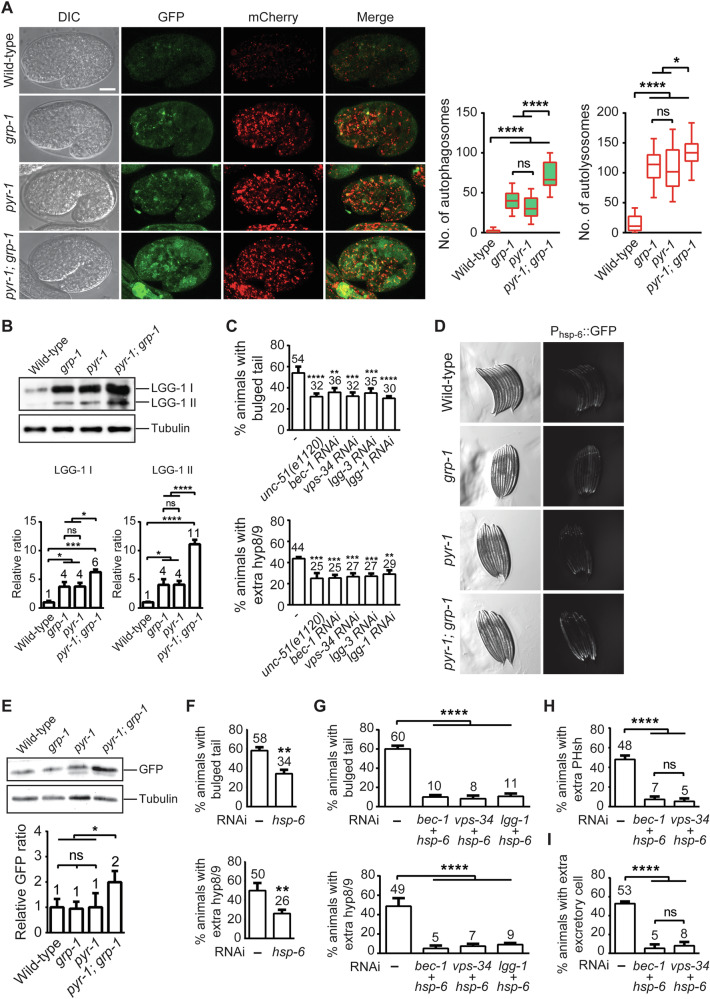
Loss of *pyr-1* induces autophagy and *hsp-6* expression to protect cell from death. **A** DIC and fluorescence images of embryos with the indicated genotypes. All strains carry the *sqIs11[P*_*lgg-1*_*::mCherry::gfp::lgg-1]* transgene. Scale bar: 10 µm. Autophagesomes (mCherry and GFP colocalization) and autolysosomes (mCherry only) were quantified and are presented as box-and-whisker plots (*n* ≥ 16). * indicates *P* < 0.05 and **** *P* < 0.0001 (one-way ANOVA with Tukey’s multiple comparisons test). ns indicates no statistical difference (*P* > 0.05). **B** Western blot analysis of the *adIs2122[P*_*lgg-1*_*::gfp::lgg-1]* transgene in the indicated genetic backgrounds using a GFP antibody. Quantification of LGG-1 I and LGG-1 II was performed from four independent experiments, with each result first normalized to tubulin and then to the wild-type. **C** Percentages of *pyr-1(tp12); grp-1(gm350)* double mutants with the *unc-51(e1120)* mutation or treated with the indicated RNAi displaying either the bulged tail phenotype or extra hyp8/9 cell marker *arIs99[Pdpy-7::2xnls::yfp]*. The RNAi was mediated by microinjection of dsRNA. Data are presented as mean ± SD from three independent experiments (*n* ≥ 30 animals per experiment). ** indicates *P* < 0.01 and *** *P* < 0.001 (one-way ANOVA with Tukey’s multiple comparisons test). **D** DIC and fluorescence images of animals carrying the *zcIs13[P*_*hsp-6*_*::gfp]* transgene in the indicated genotypes. **E** Western blot analysis of the *zcIs13[Phsp-6::gfp]* transgene in the indicated genetic backgrounds using a GFP antibody. Quantification of GFP was performed from three independent experiments, with each result first normalized to tubulin and then to the wild-type. * indicates *P* < 0.05 (one-way ANOVA with Tukey’s multiple comparisons test). ns indicates no statistical difference (*P* > 0.05). **F**, **G** Percentages of *pyr-1(tp12); grp-1(gm350)* mutants injected with the indicated dsRNAs displaying either the bulged tail phenotype or extra hyp8/9 cell marker *arIs99[Pdpy-7::2xnls::yfp]*. Data are presented as mean ± SD from three independent experiments (*n* ≥ 30 animals per experiment). ** indicates *P* < 0.01 (two-tailed *t* test) (**F**) and **** indicates *P* < 0.0001 (one-way ANOVA with Tukey’s multiple comparisons test) (**G**). Percentages of *pyr-1(tp12); grp-1(gm350)* mutants injected with the indicated dsRNAs displaying extra PHsh cell marker *tpEx436[Pver-1::4xnls::gfp]* (**H**) or excretory cell marker *nIs434[Ppgp-12::gfp]* (**I**). Data are presented as mean ± SD from three independent experiments (*n* = 50 animals per experiment). **** indicates *P* < 0.0001 (one-way ANOVA with Tukey’s multiple comparisons test).

Depletion of essential metabolites triggers significant cellular stress responses, prominently activating autophagy as a key adaptive pathway that facilitates the recycling of cellular components to replenish nutrients and maintain metabolic homeostasis [38, 39]. To assess if autophagy is activated in *pyr-1(tp12)* and *grp-1(gm350)* mutant backgrounds in response to UMP depletion, we used the dual fluorescent transgene *sqIs11[P*_*lgg-1*_*::mcherry::gfp::lgg-1]* to monitor both autophagosomes and autolysosomes [40, 41]. Autophagosomes were identified as puncta positive for both GFP and mCherry fluorescence, while autolysosomes were detected only in the mCherry channel due to quenching of GFP in the acidic lysosomal environment. We observed a significant increase in the number of autophagosomes and autolysosomes in *pyr-1(tp12)* and *grp-1(gm350)* embryos compared to wild-type embryos. An even higher accumulation of autophagosomes and autolysosomes was observed in the *pyr-1(tp12); grp-1(gm350)* double mutants (Fig. 5A). These findings indicate that mutations in *pyr-1* and *grp-1* elevated autophagy level with the double mutant exhibiting a more pronounced effect.

In *C. elegans*, LGG-1, the ortholog of mammalian LC3, is a key protein involved in autophagy, which marks autophagosome formation and enables the sequestration and degradation of cellular components [42]. We also investigated the accumulation of LGG-1 cleavage products LGG-1 I and LGG-1 II as changes in LGG-1 II levels correlate closely with the number of autophagosomes in a cell [43, 44]. Embryos expressing the transgene *adIs2122[lgg-1p::gfp::lgg-1]* in *pyr-1(tp12)* and *grp-1(gm350)* mutant backgrounds substantially accumulated LGG-1 I and LGG-1 II compared to wild-type, and these effects were further enhanced in the double mutant (Fig. 5B), consistent with the observations made using the fluorescent transgene *sqIs11[Plgg-1::mcherry::gfp::lgg-1]*.

To further investigate if autophagy plays a role in preventing cell death in *pyr-1(tp12); grp-1(gm350)* double mutants, we knocked down key autophagy components in these mutants and analyzed their tail morphology and the presence of extra hyp8/9 cells. Using the autophagy mutant *unc-51(e1120)* and RNAi-mediated knockdown of autophagy genes essential for autophagosome formation (e.g. *bec-1*, *vps-34*, *lgg-3*, and *lgg-1*), we observed a significant reduction in the percentage of *pyr-1(tp12); grp-1(gm350)* double mutants exhibiting the bulged tail phenotype and extra hyp8/9 cells (Fig. 5C). These findings show that *pyr-1* and *grp-1* mutations promote autophagy in a partially redundant manner and autophagy serves as a protective mechanism against PCD and cell fate transformation of ABpl/rpppapp in the *pyr-1*;*grp-1* double mutants.

### UMP imbalance affects the coordination of autophagy and mitochondrial stress response

Mitochondria play a central role in regulating PCD [45, 46]. Several lines of evidence suggest a functional link between pyrimidine biosynthesis and mitochondrial regulation of apoptosis. For example, reductions in mitochondrial DNA content [47, 48] and alterations in mitochondrial dynamics—such as those caused by inhibition of dihydroorotate dehydrogenase (DHODH), a mitochondrial enzyme essential for UMP biosynthesis [49]—can suppress apoptosis in mammalian systems. In addition, activation of the mitochondrial unfolded protein response (UPR^mt^), which helps maintain mitochondrial function under metabolic stress (e.g., NAD^+^ depletion or impaired mitochondrial transcription or translation [50, 51]), has also been shown to suppress apoptosis [52]. Notably, HSP6, a key mediator of UPR^mt^, has been implicated in anti-apoptotic responses [53, 54]. Based on these connections, we investigated whether mitochondrial DNA content, morphology, and UPR^mt^ were altered in *pyr-1(tp12); grp-1(gm350)* double mutants, potentially contributing to their abnormal cell survival phenotype. While we did not detect significant changes in mitochondrial DNA content or mitochondrial morphology (Supplementary Fig. S4), we observed a significant induction of *P*_*hsp-6*_*::GFP* reporter expression specifically in the double mutants, but not in the wild-type or single mutants, indicating that mitochondrial stress responses were activated [55, 56] (Fig. 5D, E). The cell death defects, including the bulged tail phenotype and the formation of extra hyp8/9 cells, associated with *pyr-1(tp12); grp-1(gm350)* double mutants were significantly alleviated when *hsp-6* was knocked down in the double mutant (Fig. 5F). These findings suggest that HSP-6 promotes cell survival in *pyr-1(tp12); grp-1(gm350)* double mutants. Remarkably, simultaneous knockdown of *hsp-6*, along with autophagy components such as *bec-1*, *vps-34*, or *lgg-1*, almost completely rescued the extra hyp8/9 cells phenotype in *pyr-1(tp12); grp-1(gm350)* mutants (Fig. 5G). Similarly, combined knockdown of *hsp-6* and either *bec-1* or *vps-34* also rescued the abnormal extra PHsh and excretory cell phenotype (Fig. 5H, I). These results suggest the collaborative roles of autophagy and HSP-6 in protecting cells from PCD across multiple cell lineages in the *pyr-1(tp12); grp-1(gm350)* mutants.

## Discussion

PCD is a fundamental process essential for development, tissue homostasis, and the removal of unnecessary or damaged cells. In cells destined to die, the BH3-only protein EGL-1 is transcriptionally activated and binds to the BCL-2 protein CED-9. This interaction induces a conformational shift that releases CED-4 and activates the caspase CED-3, which executes the cell death [57, 58]. Thus, *egl-1*, *ced-9*, and *ced-4* function in the activation of PCD, and *ced-3* carries out its execution. The roles of these proteins are highly conserved between *C. elegans* and humans [59, 60], making *C. elegans* a valuable model for studying PCD mechanisms in broader biological and disease contexts. In our study, we demonstrated synergistic interactions between *grp-1* and *ced-3* in regulating PCD and cell differentiation within multiple cell lineages in *C. elegans*. In *grp-1; ced-3* double mutants, a bulged tail phenotype arises from the inappropriate survival and differentiation of cells that normally undergo PCD in the ABpl/rpppap lineage, leading to the production of additional hyp8/9 and PHsh cells. Through a genetic screening for PCD-related mutations in the sensitized *grp-1* mutant background, we identified *pyr-1* as a critical regulator of PCD through UMP biosynthesis. Genetic analysis of UMP metabolic pathways, combined with supplementation experiments, confirmed that UMP is the critical metabolite for *pyr-1*-mediated PCD. Furthermore, we showed that autophagy and *hsp-6* expression are induced in the *pyr-1; grp-1* mutants. Autophagy likely functions to recycle cellular and/or metabolic components and maintain their homeostasis, while *hsp-6* induction may mitigate mitochondrial stress. Notably, simultaneous knockdown of autophagy components and *hsp-6* in *pyr-1; grp-1* mutants nearly rescued the bulged tail phenotype, demonstrating that these pathways act in concert to promote cell survival under combined metabolic and cellular stress conditions resulted from *pyr-1* and *grp-1* double mutations.

Stress responses allow cells to adapt to metabolic deficiencies, maintain homeostasis, and survive under nutrient-deprived conditions [38, 39, 61, 62]. In this context, autophagy provides an emergency recycling mechanism critical for cell survival during periods of metabolic stress by degrading and recycling cellular components [38, 39]. BEC-1, the *C. elegans* ortholog of Beclin-1, serves as a link between apoptosis and autophagy [63]. BEC-1 interacts with the anti-apoptotic protein CED-9, and its loss triggers CED-3/caspase-dependent apoptosis [63]. Interestingly, the loss of VPS-34, which interacts with BEC-1 and is essential for autophagy, has no effect on apoptosis [63]. It is likely that BEC-1 forms two distinct functional complexes: one with VPS-34 to regulate autophagy and another with CED-9 to influence apoptosis [63]. Additionally, BEC-1 and other autophagy-related genes are required for the clearance of apoptotic cell [64]. In our study, knockdown of *bec-1* significantly reduced cell death defects. BEC-1 may act as a mediator of the autophagic response to UMP deficiency, integrating signals from metabolic stress pathways and apoptotic regulators. This multifunctional role highlights that BEC-1 plays context-dependent roles, adapting its interaction to cellular needs.

Compared to autophagy, the relationship between mitochondrial stress response and apoptosis remains less defined. Although the loss of either *grp-1* or *pyr-1* alone has little impact on *hsp-6* expression, their combined loss induces the mitochondrial chaperone *hsp-6*, further aggravating the PCD defect. HSPA9, the human ortholog of HSP-6, has been implicated in apoptosis [53, 54]. Knockdown of HSPA9 induces apoptosis in human hematopoietic progenitor cells in a TP53-dependent manner [53], while its overexpression inhibits apoptin-induced apoptosis in HepG2 cells by altering apoptin distribution [54]. These findings align with our results, suggesting that HSP-6/HSPA9 functions as a protector against apoptosis. Our findings indicate that UMP deficiency triggers both autophagy and *hsp-6* expression as compensatory mechanisms to mitigate metabolic stress, particularly in the context of *grp-1* mutations. These results expand our understanding of how autophagy and *hsp-6* contribute to cellular homeostasis and provide insights into the broader impact of metabolic dysfunction on cell survival and developmental processes.

UMP is a pivotal metabolite in this protective response against metabolic stress. It acts as a key product in the de novo pyrimidine synthesis pathway: UMP and its downstream nucleotide metabolites are essential for the synthesis of DNA, RNA, lipids, and carbohydrates [17]. UMP depletion may signal a critical scarcity of resources, potentially triggering protective mechanisms. Recent studies have identified nucleotide-sensing receptors in mammalian cells that regulate metabolism and stress responses [65, 66]. It is plausible that cellular UMP levels are monitored by specific sensors or proteins responsive to UMP availability. Future studies could explore potential UMP-deficiency sensors that may influence cell death in response to metabolic shifts.

Human CAD has been identified as a substrate of caspase 3 and is cleaved during apoptosis induced by staurosporine or doxorubicin [16]. Similarly, we found that PYR-1 is cleaved by the CED-3 protease in vitro and in vivo (Supplementary Fig. S5). However, CRISPR-Cas9-mediated mutagenesis of the cleavage site in PYR-1 does not affect PCD (Supplementary Fig. S5), showing that PYR-1 mediates PCD through a mechanism independent of its cleavage. Our work revealed that PYR-1 mediates PCD through the biosynthesis of UMP. Whether CAD, like PYR-1, contributes to PCD via pyrimidine biosynthesis requires further studies.

Oncogene-driven cancers often exhibit elevated pyrimidine de novo synthesis [67]. Emerging cancer therapies targeting key enzymes in this pathway, such as CAD and DHODH inhibitors, show potential to disrupt tumor growth [67–69]. However, our findings suggest potential limitations of these strategies: insufficient UMP levels can impair apoptosis and activate stress responses, including autophagy and mitochondrial chaperon-mediated mechanisms, which promote cell survival. These adaptive mechanisms may undermine the efficacy of therapies that suppress UMP synthesis. It is worth investigating whether the loss of CAD in human cells triggers a self-protective mechanism, such as enhanced autophagy or HSP-6-mediated stress responses. Unraveling these pathways could provide critical insights into how metabolic stress is managed in cancer cells and inform strategies to overcome resistance. Such understanding may help optimize pyrimidine metabolism-targeted therapies, potentially by combining them with inhibitors of autophagy or mitochondrial UPR to improve therapeutic efficacy.

In conclusion, our study elucidates a complex interplay between PCD, metabolic regulation, and stress responses in maintaining developmental integrity. The combined role of *grp-1* with *ced-3* and *pyr-1* in orchestrating PCD and ensuring cellular homeostasis provides insights into how metabolic and cell death pathways converge to support proper tissue morphogenesis. Future research exploring the molecular interactions between UMP and PCD machinery and the broader physiological implications of this interaction will open exciting avenues in health and disease.

## Supplementary information

Supplementary Figures

Supplementary Information

Western Blot raw

## Data Availability

All data generated or analyzed during this study are included in this published article and its Supplementary Information files.
