# Supplementary material for: Uridine 5’-monophosphate (UMP) synthesis connects nucleotide metabolism to programmed cell death in C. elegans
Source: Cell Death Differ. 2025 Sep 3;33(1):25–37. doi: 10.1038/s41418-025-01564-x (PMC12811246; doi:10.1038/s41418-025-01564-x)
Supplement: Supplementary file 1 — Supplementary Figures [file 41418_2025_1564_MOESM1_ESM.docx]

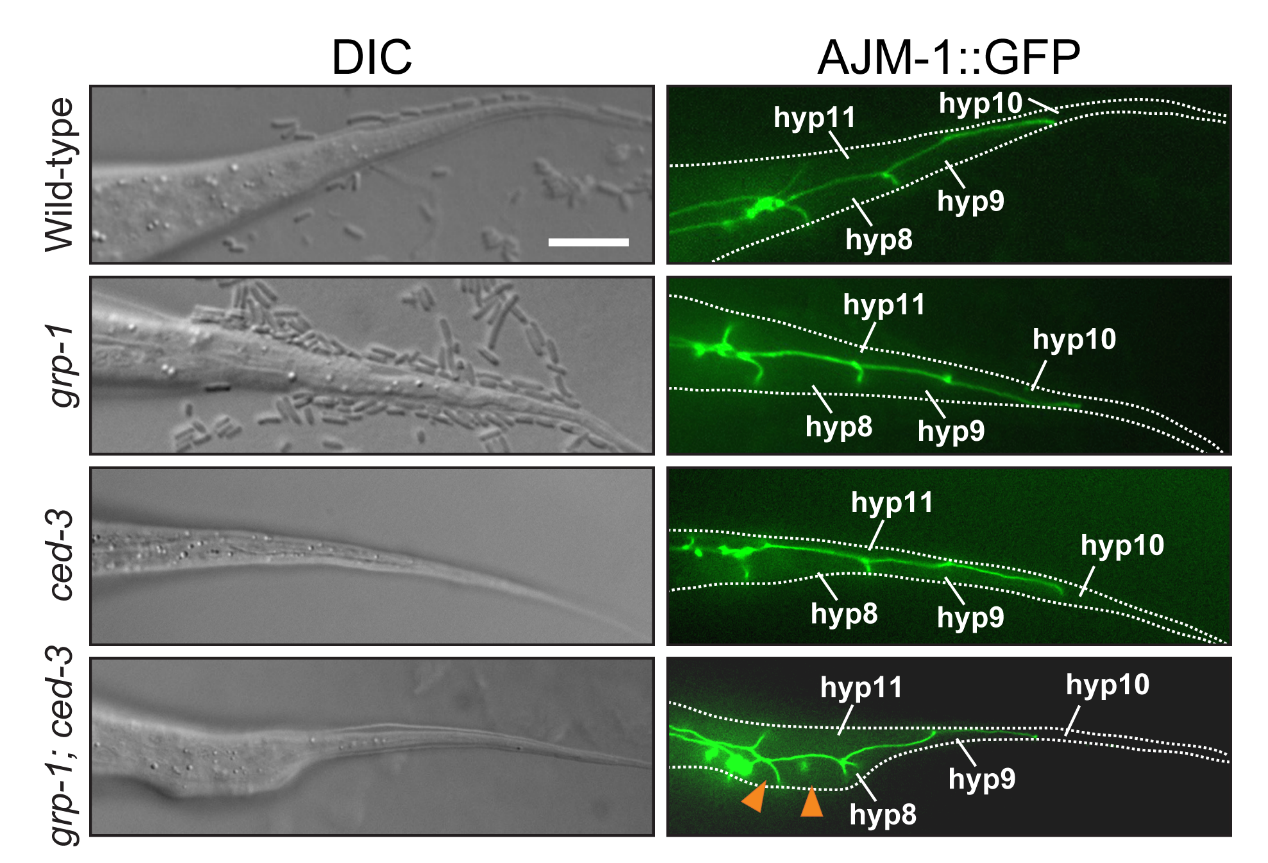


**Fig. S1 The *grp-1(gm350); ced-3(n717)* mutant possesses extra hyp8/9 cells in the bulged tail region.** DIC and fluorescence images of L3-stage animals carrying the adherens junction marker *jcIs1[ajm-1::gfp]* in different genetic backgrounds. White lines point to the hypodermal cells hyp8, hyp9, hyp10, and hyp11. The orange arrowheads highlight two additional hyp8/9 cells in the *grp-1;ced-3* double mutant. Scale bar: 10 µm.


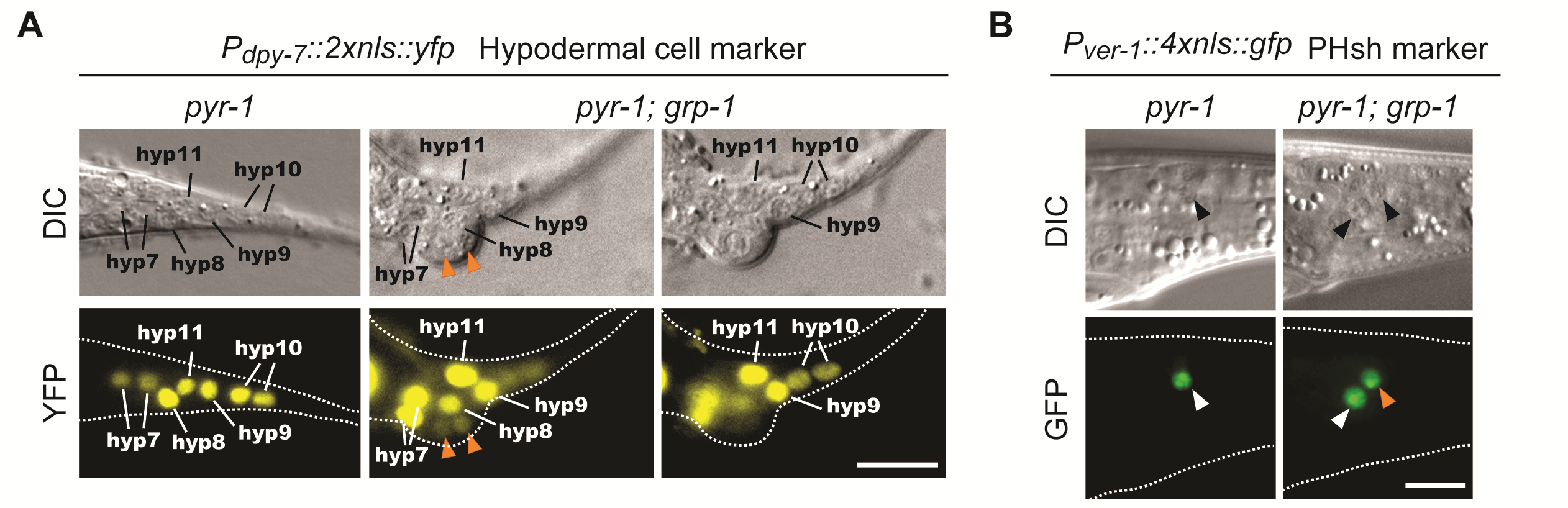


**Fig. S2 Extra hyp8/9 cells contribute to the bulged tail phenotype in *pyr-1(tp12); grp-1(gm350)* mutants.** DIC and fluorescence images of animals carrying the tail hypodermal cell marker *arIs99[P_dpy-7_::2xnls::yfp]* (**A**) or the PHsh marker *tpEx436[P_ver-1_::4xnls::gfp]* (**B**) in the indicated genetic background. Orange arrowheads indicate extra hyp8/9 (**A**) or PHsh (**B**). Two focal planes of *tp12; grp-1; arIs99* animals are shown to present tail hypodermal cells. Scale bar: 10 µm. Alleles used: *pyr-1(tp12)* and *grp-1(gm350)*.


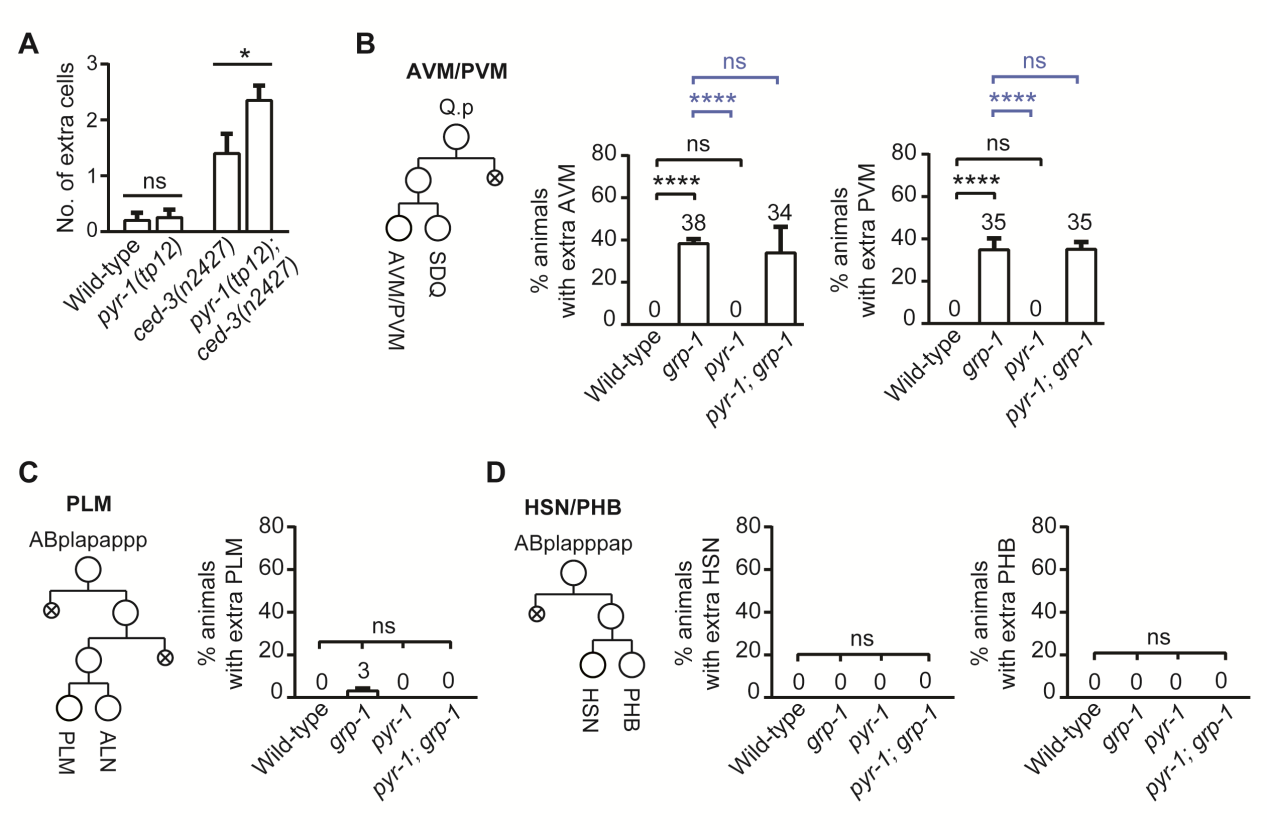


**Fig. S3 The *pyr-1(tp12)* mutation results in an increase number of extra pharyngeal cells but does not affect the number of AVM, PVM, PLM, HSN, and PHB neurons in the *grp-1(gm350)* background. A** Analysis of surviving cells in the pharyngeal region (n=20) for each genotype. Data are presented as mean ± SD (n = 20 animals per experiment). * indicates P < 0.05 (two-tailed t test). **B-D** Quantification of animals with extra AVM, PVM (**B**), PLM (**C**), HSN, and PHB (**D**) neurons across different genotypes. Neurons were visualized using the following transgenic markers: *zdIs5[P_mec-4_::gfp]* for AVM, PVM, and PLM; *mgIs71[tph-1::gfp]* for HSN; and *gmIs12[P_srb-6_::gfp]* for PHB. Data present the mean ± SD from three independent experiments (n≥ 47 per experiment). **** indicates *P* < 0.0001 (one-way ANOVA with Tukey’s multiple comparisons test). ns indicates no statistical difference (*P* > 0.05).


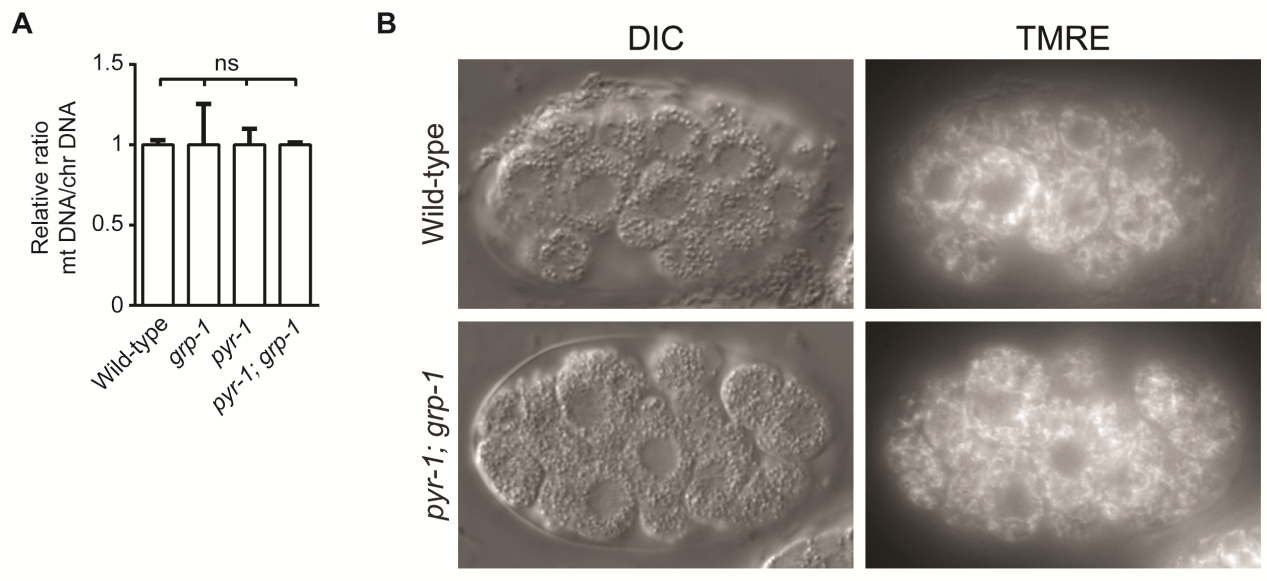


**Fig. S4 The *pyr-1(tp12); grp-1(gm350)* mutation does not affect the mitochondrial DNA content and morphology. A** Mitochondrial DNA copy number was assessed by real-time quantitative PCR using amplification of an mtDNA fragment (*cyb*) normalized to a nuclear genomic fragment (*K01H12.2*), as previously described [17]. Data represent the mean ± SD from three independent experiments. No statistically significant difference were observed among genotypes (*P* > 0.05, ns; one-way ANOVA with Tukey’s multiple comparisons test). **B** DIC and fluorescence images of early embryos from the indicated genotypes following TMRE staining. *C. elegans* hermaphrodites were cultured overnight at 20 °C on NGM plates supplemented with 5 μM TMRE. Embryos were obtained by dissecting gravid hermaphrodites to release embryos from the gonads [19] and imaged using fluorescence microscopy.


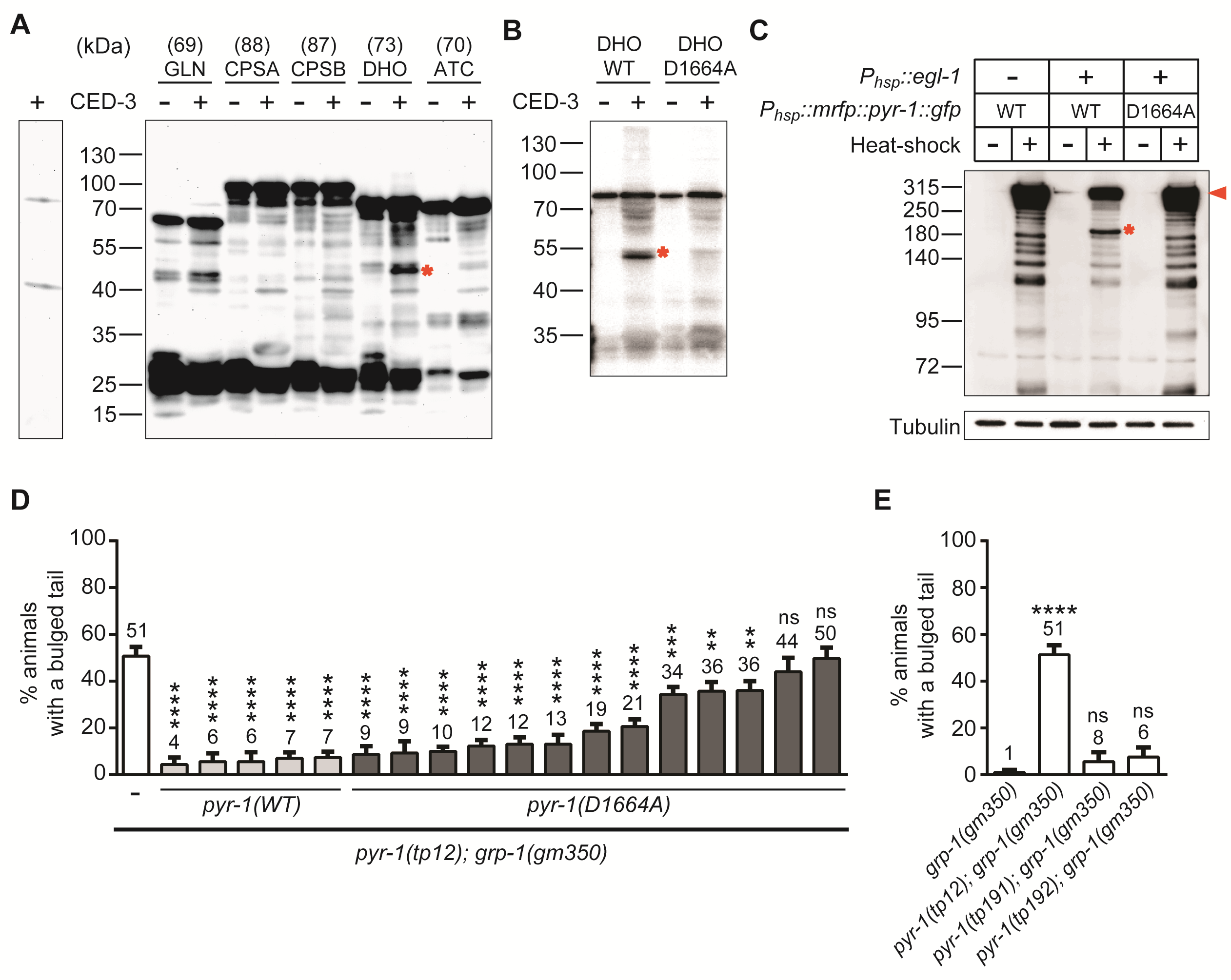


**Fig. S5 CED-3-mediated cleavage of PYR-1 does not contribute to the bulged tail phenotype in the *grp-1(gm350)* background. A** CED-3 cleaves PYR-1 at the DHO domain *in vitro*. GST-tagged GLN, CPS-A, CPS-B, DHO, and ATC domains, along with His-tagged CED-3, were purified from bacteria as described in MATERIALS AND METHODS. Each domain was incubated with or without CED-3 and analyzed via western blot using an anti-GST antibody. The predicted protein sizes are indicated in parentheses. The red asterisk marks a cleaved fragment observed in the DHO domain following CED-3 incubation. **B** CED-3 cleaves the DHO domain at the 1664^th^ aspartic acid residue of PYR-1 *in vitro*. CED-3, an aspartate-specific cysteine protease, cleaves substrates at aspartic acid residues. Based on the cleaved fragment size (40-55 kDa), the cleavage site was identified as the 1664^th^ aspartic acid in PYR-1 (197^th^ in the DHO domain). Wild-type (WT) and point-mutated (D1664A) DHO domains were incubated with or without CED-3 and analyzed via western blot using an anti-GST antibody. The red asterisk indicates a cleaved fragment present in the DHO(WT) domain but absent in the DHO(D1664A) domain. **C** CED-3 cleaves PYR-1 at the 1664^th^ aspartic acid *in vivo*. Transgenic animals carrying *P_hsp_::mrfp::pyr-1::gfp* (lane 1 and 2), *P_hsp_::mrfp::pyr-1::gfp* with *P_hsp_::egl-1* (lane 3 and 4), or *P_hsp_::mrfp::pyr-1(D1664A)::gfp* with *P_hsp_::egl-1* (lane 5 and 6) were generated as described in MATERIALS AND METHODS. Heat-shock treatment (33°C for 1 hour, followed by recovery at 20°C for 2 hours) induced the expression of PYR-1 or PYR-1 with EGL-1, which activates CED-3. Lysates were immuneprecipitated and analyzed via western blot using an anti-GFP antibody (detail in MATERIALS AND METHODS). The red arrowhead marks full-length mRFP::PYR-1::GFP (296 kDa) after heat-shock, while the asterisk marks the N-terminal cleavage fragment (209 kDa) produced by CED-3-mediated cleavage at the 1664^th^ aspartic acid residue in PYR-1. The C-terminal cleavage fragment (87 kDa) was undetected, likely due to rapid degradation. A 1/25X lysate sample was loaded as an internal control and analyzed with an anti-tubulin antibody. **D** Disruption of the CED-3 cleavage site does not affect the rescuing activity of PYR-1. Percentages of animals with a bulged tail phenotype for the indicated genotypes are presented as mean ± SD from three independent experiments (n = 50 animals per experiment). Five independent lines of *pyr-1(tp12); grp-1(gm350)* mutants carrying wild-type PYR-1 (WT) and 13 lines carrying point-mutated PYR-1 (D1664A) were analyzed. Each line was compared to *pyr-1(tp12); grp-1(gm350)* mutants (-). **E** Disruption of the CED-3 cleavage site in PYR-1 does not cause a bulged tail phenotype. Percentages of animals with a bulged tail phenotype for indicated genotypes are presented as mean ± SD from three independent experiments (n = 50 animals per experiment). Two independent *pyr-1(D1664A)* lines, *tp191* and *tp192*, were generated via CRISPR genomic editing and crosses into *grp-1(gm350)* mutants. Each genotype was compared to *grp-1(gm350)* mutants.
