# Supplementary material for: Uridine 5’-monophosphate (UMP) synthesis connects nucleotide metabolism to programmed cell death in C. elegans
Source: Cell Death Differ. 2025 Sep 3;33(1):25–37. doi: 10.1038/s41418-025-01564-x (PMC12811246; doi:10.1038/s41418-025-01564-x)
Supplement: Supplementary file 2 — Supplementary Information [file 41418_2025_1564_MOESM2_ESM.docx]

**SUPPLEMENTARY INFORMATION**

**MATERIALS AND METHODS**

**C. elegans strains**

C. elegans strains were maintained at 20°C as described previously [1]. The N2 Bristol strain was used as the wild-type strain. The mutations used were as follows: LGI, *ced-1(e1735)*, *ced-12(bz187)*; LGII, *pyr-1(tp12, ΔOMPDC, tp191, tp192)* (this study), *pyr-1(cu8)*; LGIII, *ced-9(n1950)*, *ced-4(n1162)*, *unc-119(ed3)*, *pha-1(e2123)*; LGIV, *ced-3(n717, n2427, n2923)*, *jcIs1[ajm-1::gfp]*; LGV, *egl-1(n1084n3082)*, *zcIs13[P_hsp-6_::gfp]*; LGX, *ced-8(n1891)*. Strain GS3798 carrying *arIs99[P_dpy-7_::2xnls::yfp]*, MAH215 carrying *sqIs11[P_lgg-1_::mcherry::lgg-1::gfp]*, and DA2123 carrying a*dIs2122[P_lgg-1_::gfp::lgg-1]* are not mapped to specific chromosomes. Strain NG4590 carrying *zdIs5[P_mec-4_::gfp]*, NG5055 carrying *mgIs71[tph-1::gfp]*, NG2693 carrying *gmIs12[P_srb-6_::gfp]*, and the *grp-1(gm350)* mutant were kindly provided by Gian Garriga [2]. Strain MT20084 carrying *nIs434[P_pgp-12_::gfp]* was kindly provided by H. Robert Horvitz [3]. The transgene *bcIs37[P_egl-1_::his-24::gfp]* was kindly provided by Barbara Conradt [4]. The *cnt-1(tm2313)*, *C47B2.2(tm2030)*, and *B0001.4(tm2740)* mutants were generated and provided by the National Bioresource Project in Japan. The *cps-6(ok1718)* and *pyr-1(ok2391)* mutants were provided by the *C. elegans* Knockout Consortium [5]. The CB4856 strain was used for SNP mapping [6].

**Transgenic animals**

Transgenic animals were generated through germline transformation as described previously [7]. To generate *P_ver-1_::4xnls::gfp* transgenic worms, wild-type worms were injected with 50 ng/µl *P_ver-1_::4xnls::gfp*, along with 50 ng/µl of the co-injection marker *sur-5::gfp*. To generate *P_lin-26_::mcherry* and *P_lin-26_::ced-9(gf)* transgenic worms, 100 ng/µl of *P_lin-26_::mcherry* or *P_lin-26_::ced-9(gf)* was injected into *grp-1(gm350)* mutants with 50 ng/µl of the co-injection marker *rol-6*. For rescue experiments, 30 ng/µl *pyr-1* was injected into *pyr-1(tp12); grp-1(gm350)* mutants together with 50 ng/µl of *sur-5::gfp* as the co-injection marker. To generate transgenic animals carrying *P_hsp_::mrfp::pyr-1::gfp* or *P_hsp_::mrfp::pyr-1::gfp* plus *P_hsp_::egl-1*, 50 ng/µl *P_hsp_::mrfp::pyr-1::gfp* with or without 50 ng/µl *P_hsp_::egl-1* was injected into *unc-119(ed3); egl-1(n1084n3082)* mutants with 30 ng/µl of the co-injection marker *unc-119(+)*. To perform rescue experiments, 50 ng/µl *P_pyr-1_:: pyr-1 cDNA::gfp* or 50 ng/µl *P_pyr-1_:: pyr-1 cDNA(D1664A)::gfp* was injected into *pyr-1(tp12); grp-1(gm350)* mutants with 50 ng/µl of the co-injection marker *rol-6*. The *umps-1(ΔOMPDC)* and *grp-1(gm350) umps-1(ΔOMPDC)* mutants were generated via CRISPR as previously described [8], using a mixture containing the following components: 0.25 µg/µL Cas9, 0.02 µg/µL tracrRNA, 0.02 µg/µL crRNA, 0.11 µg/µL single-stranded oligodeoxynucleotides donor, and 40 ng/µL *rol-6*. F1 progeny expressing the co-injection marker were cloned and F2 progeny were screened for homozygosity, with validation through sequencing. *tp191* and *tp192* were generated via an alternative CRISPR method [9], using a mixture containing the following components: 60 ng/µL pJW1285 (*pha-1* targeting CRISPR/Cas9), 60 ng/µL pYW1738 (*pyr-1* targeting CRISPR/Cas9), 50 ng/µL *pha-1* repair oligo, 50 ng/µL *pyr-1* repair oligo, and 2 ng/µL pCFJ90 (*P_myo-2_::mcherry*). Viable F1 progeny were cloned and F2 progeny were screened for homozygosity, with validation through sequencing.

**Mutant screen and cloning of *pyr-1(tp12)***

The *grp-1(gm350)* mutants were mutagenized with 75 mM ethylmethanesulfonate (M0880, Sigma-Aldrich) for 4 hours at 20°C. Approximately 12,000 F2 progeny were screened for a bulged tail phenotype under a dissecting microscope. The *pyr-1* mutation was identified as the causal mutation through a combination of techniques, including single nucleotide polymorphism (SNP) mapping using the natural *C. elegans* CB4856 strain, complementation test, fosmid rescue, whole genome sequencing, and candidate gene (*pyr-1*) analysis. In SNP mapping, the CB4856 strain carrying the *grp-1(gm350)* mutation, CB4856(*grp-1/gm350*), was generated. *tp12; grp-1(gm350)* mutants were crossed to CB4856(*grp-1/gm350*), and F2 progeny with a bulged tail phenotype were analyzed for SNP, including pkP2140, pkP2103, pkP2107, pkP2069, and uCE2131. No recombination was detected at pkP2107, positioning *tp12* between pkP2103 (-6.21 cM) and pkP2069 (2.65 cM). Four deficiencies located between -6.21 and 2.65 cM (*mnDf30*, *mnDf66*, *mnDf62*, and *mnDf68*) were selected for complementation test. These deficiencies were first crossed to *grp-1(gm350)* mutants, and the resulting double mutant were crossed to *tp12; grp-1(gm350)* mutants. The average percentages of F1 progeny with the bulged tail phenotype were: *tp12/tp12*: 50%, *tp12/mnDf30*: 15%, *tp12/mnDf66*: 40%, *tp12/mnDf62*: 10%, and *tp12/mnDf68*: 62%. These results positioned *tp12* between 0.83 and 1.02 cM. Within this region, three fosmids (WRM0611bE04, WRM0638bB03, and WRM0639aD10) were selected for rescue experiments. Each fosmid (30 ng/µL) was injected to *pyr-1; grp-1(gm350)* mutants along with 50 ng/µl *sur-5::gfp* as a co-injection marker. The average percentages of transgenic animals exhibiting the bulged tail phenotype were: WRM0611bE04: 40%, WRM0638bB03: 18%, and WRM0639aD10: 27%. These results positioned *tp12* between 8643076 and 8657635 bp, a region containing two genes: *D2085.7* and *pyr-1*. Whole genome sequencing revealed that only *pyr-1* contained an exonic mutation. Subsequent rescue experiments using a PCR product of genomic *pyr-1* containing 3.8 kb upstream of the start codon and 2.1 kb downstream of the stop codon confirmed that *tp12* is an allele of *pyr-1*.

**RNA interference (RNAi)**

RNAi was performed by microinjecting double-stranded RNA as described previously [10]. The double-stranded RNA was synthesized via *in vitro* transcription from templates amplified from the Ahringer RNAi library [11] with T7 primers or from PCR products generated from worms with specific primers, detailed in the Supplementary information.

**Cell death assays**

The percentage of animals displaying a bulged tail phenotype was scored at the L4 stage under a dissecting microscope. Cell corpses were counted at specified developmental stages and extra cells in the anterior pharynx of L4 hermaphrodites were scored using Nomarski optics [12, 13]. To analyze ABpl/rpppapp and ABplpappap cell deaths, embryos were mounted on 4% agar pads and recorded using four-dimensional DIC microscopy [14]. To quantify hyp8/9, PHsh, and excretory cells, transgenes *arIs99[Pdpy-7::2xnls::yfp]*, *tpEx436[Pver-1::4xnls::gfp]*, and *nIs434[Ppgp-12::gfp]* were used, with fluorescent cells counted by fluorescence microscopy.

**Molecular biology**

Standard methods of cloning, sequencing, and PCR were used. The *lin-26* promoter for *P_lin-26_::mCherry* was PCR- amplified and inserted into the HindIII site of pYW806, a modified pPD95.75 vector where *gfp* was replaced by *mcherry*. For *P_lin-26_::ced-9(gf)*, the *lin-26* promoter was amplified by PCR from wild-type worms, and *ced-9(gf)* cDNA was amplified from the cDNA library generated from *ced-9(n1950)* mutants and then inserted into the HindIII and KpnI sites of pPD95.75, respectively. To generate *P_ver-1_::4xnls::gfp*, the *ver-1* promoter was amplified and inserted into the PstI and BamHI sites of pPD122.56. Genomic *pyr-1* was amplified from WRM0638bB03 and cloned into a pENTR™/D-TOPO® vector. To generate *P_hsp_::mrfp::pyr-1::gfp*, *pyr-1* cDNA and *mrfp* were amplified and inserted into pPD95.75 by KpnI and XmaI/PpuMI, respectively. The construct was then treated with XmaI and AflII and the *mrfp::pyr-1::gfp* fragment was then inserted into pPD49.83 by XmaI and AflII. To generate *P_pyr-1_::pyr-1::gfp*, *pyr-1* promoter (3.8kb upstream of start codon) and cDNA were amplified and inserted into pPD95.75 by PstI and KpnI, respectively. The point-mutated *pyr-1(D1664A)* was generated using site-directed mutagenesis. Primer sequences are listed in the Supplementary information.

**Uracil and uridine supplementation**

Uracil and uridine (Sigma-Aldrich) were dissolved into NGM (liquid) immediately before plate preparation. Plates were seeded with *E. coli* OP50 and UV-irradiated in a laminar flow cabinet for at least 4 hours to eliminate live bacteria [15]. Overnight cultures confirmed bacterial inactivation. L4 worms were placed on plates and transferred every 24hr, with F1 progeny analyzed at the L4 stage.

**Western blot**

Twenty worms were lysed in 20µL of 2X RIPA buffer, heated at 95°C for 5 min with loading dye, then separated on 10% SDS-PAGE gels and transferred to the PVDF membrane. Blots were probed with anti -GFP (ab290, Abcam, 1:1000) and α-tubulin (12G10, DSHB, 1:2000) antibodies.

**Imaging and quantification of autophagy**

1.5-fold embryos of wild-type or specified mutants carrying the transgene *sqIs11[P_lgg-1_::mCherry::gfp::lgg-1]* were mounted on 4% agar pads containing M9 buffer and imaged using a Zeiss LSM 780 Confocal Microscope. Images were captured beginning with the plane of largest area as the focal point, followed by additional images taken at 2 µm intervals above and below this plane, with two images acquired in each direction. The numbers of autophagosomes (puncta displaying both GFP and mCherry signals) and autolysosomes (puncta with mCherry signal only) were quantified for these five images using Image J with consistent settings and presented as the average of the five images.

**CED-3 cleavage analysis of PYR-1 *in vitro* and *in vivo***

For *in vitro* analysis of CED-3-mdiated cleavage, individual domains of PYR-1 were amplified and inserted into the TA vector, then cloned into the pGEX 4t-1 expression vector (primer sequences are listed in the Supplementary information). The construct *pET-3a::ced-3::flag::his* was generously provided by Ding Xue [16]. Expression of GST-tagged PYR-1 domains and CED-3 was induced in *Escherichia coli* strain BL21 carrying the respective plasmids. Proteins were purified using glutathione Sepharose (Amersham Biosciences) and Ni-NTA Agarose (QIAGEN) following the manufacturer's protocol. GST-tagged PYR-1 domains were incubated with purified CED-3 as described previously [16, 17]. The resulting cleavage products were analyzed by western blotting with an anti-GST antibody (27-4577-01, Amersham Biosciences, 1:2000). For *in vivo* analysis, transgenes were injected into *unc-119(ed3); egl-1(n1084n3082)* mutants, which lack activated CED-3 but can restore its activation when *P_hsp_::egl-1* is expressed. Transgenic animals were incubated at 33°C for 1 hour and then allowed to recover at 20°C for 2 hours to induce protein expression. Lysates (500 µg) were harvested and immuneprecipitated using an anti-GFP antibody (ab290, Abcam) and the Pierce^TM^ Crosslink Magnetic IP/Co-IP Kit (88805, Thermo Scientific), following the manufacturer's protocols. Immuneprecipitated products were analyzed by western blot using the same anti-GFP antibody. Lysates (20 µg) were also analyzed as a loading control using an anti-α-tubulin antibody (12G10, DSHB, 1:2000).

**Statistical analysis**

Data in histograms are presented as mean ± standard deviation. Statistical analyses were conducted using GraphPad Prism version 10. One-way ANOVA with Tukey’s multiple comparisons test was used for all comparisons unless otherwise indicated. Significance was set at *P* < 0.05, with **P* < 0.05, ***P* < 0.01, *** *P*< 0.001, and ****P < 0.0001. ns indicates no significance (*P* > 0.05).

**Primer List**

| **Primers for construct** | |
| --- | --- |
| lin-26 promoter F | GATCAAGCTTCTCTTCTAACCATCAATTTTAG |
| lin-26 promoter R | GATCAAGCTTGGAAGAGAGGTGTGAAGGGATC |
| ced-9(gf) cDNA F | TTGGTACCATGACACGCTGCACG |
| ced-9(gf) cDNA R | GGGGTACCGACTTCAAGCTGAACATC |
| ver-1 promoter F | GGCTGCAGCAATGCACTGGAATACTCCAGAG |
| ver-1promoterR | CCGGATCCAACTCTACAAACTTTCCAATTTTTTG |
| pyr-1(U3.8D2.1) F | CACCACGGAATCACGGCCAAACAA |
| pyr-1(U3.8D2.1) R | TTCTCGGAATGCTCCCCCA |
| **Primers for dsRNA** | |
| bec-1RNAiF | CGCGCGTAATACGACTCACTATAGGGCGAATCCCATCTGATGCTCCAGTTT |
| bec-1RNAiR | CGAAATTAATACGACTCACTATAGGGAGACCCAACTGCAAGAATCGACGAA |
| vps-34RNAiF | CGCGCGTAATACGACTCACTATAGGGCGAATGTTGGATCCCTTTGCATCAC |
| vps-34RNAiR | CGAAATTAATACGACTCACTATAGGGAGACCTATGTGCCGAAATTGCAAAA |
| lgg-3RNAiF | CGCGCGTAATACGACTCACTATAGGGCGAATTTCAAACTTCCGCCATTTTACTA |
| lgg-3RNAiR | CGAAATTAATACGACTCACTATAGGGAGACCCCTCATCGTGATCGATTTTCTTA |
| lgg-1RNAiF | CGCGCGTAATACGACTCACTATAGGGCGAATTGTTATCGAATCGGTCAATCTCT |
| lgg-1RNAiR | CGAAATTAATACGACTCACTATAGGGAGACCTGACATTTTCAGACGATGACAAC |
| hsp-6RNAiF | CGCGCGTAATACGACTCACTATAGGGCGAATGCGTGATCGAAGTCTTCTCC |
| hsp-6RNAiR | CGAAATTAATACGACTCACTATAGGGAGACCGATGCAAAATGCCCTGACTT |
| T7F | GCAACCTGGCTTATCGAAATTAATAC |
| T7R | TCACGACGTTGTAAAACGACG |
| pyr-1 cDNA F | TAGGTACCATGAGAGCGACTTTGCA |
| pyr-1 cDNA R | ACGGTACCAAAAGATGCCCTCTTCC |
| pyr-1 promoter F | ATACTGCAGACGGAATCACGGCCAAAC |
| pyr-1 promoter R | GGACTGCAGACTTTCCCAATGCATTCG |
| mrfpF(XmaI) | CGCCCGGGATGGCCTCCTCCGAGG |
| mrfpR(PpuMI) | CAGGAGGACCCGAGGCGCCGGTGGAGTGG |
| GLN F | TTTGCCACCATGAGAGCGACTTT |
| GLN R | GCTTATGTGCCACTCTTTGCTTGTC |
| CPSA F | GTACCCACCATGTTCATGAATGTC |
| CPSA R | CCTTATGAAACATCATTCTCTATTCC |
| CPSB F | CTATCCACCATGTTCAATATGAAGA |
| CPSB R | GATTAGGAAGTCACACAATCGACC |
| DHO F | TAAACCACCATGGTGACTTCCAAGA |
| DHO R | AGTTAGAATGCAATCGGCGTATGAG |
| ATC F | TATACCACCATGCCTGGAGAGCTT |
| ATC R | CGTTAAAGATGCCCTCTTCCAAGA |
| **CRISPR** | |
| crRNA | GAUGAGUGCAGAUUCUGUGAGUUUUAGAGCUAUGCU |
| oligodeoxynucleotides donor | TTCTTCCGGCGCATGATTCCAAGAATTTTCTTGATGAGTGCAGACTTATGTGAGAGATTCGCGTTCCGCAAGCGGAGTTCGCACGTTTTCCACGTACGGT |
| pha-1 repair oligo | CAAAATACGAATCGAAGACTCAAAAAGAGTATGCTGTATGATTACAGATGTTCATCAAGTTATTCATAAATCATTGATAG |
| pyr-1 repair oligo | CCTGTGAAGTCTGCCCACATCATCTTTTCCTCATTGAAGAAGATCTTCCTGCTGGAATCCGTGAAGTTCGTCCACGACTCGTAAAACCAGAAGATCGTCAGGCTCTTTGGGACAACATGGAATACATT |
| **Determination of mitochondrial DNA copy number** [18] | |
| chrDNA (K01H12.2) F | CGTGAATTCAAAGGTCTGGCT |
| chrDNA (K01H12.2) R | AGTAAGCGGCACGGTAGATGA |
| mtDNA (cyb) F | CGCCCGATAGGTTAATAGCA |
| mtDNA (cyb) R | TGGCCCCATTAAAATGAAAA |

**SUPPLEMENTARY REFERENCES**

1 Brenner S. The genetics of Caenorhabditis elegans. Genetics. 1974;77:71-94.

2 Teuliere J, Cordes S, Singhvi A, Talavera K, Garriga G. Asymmetric Neuroblast Divisions Producing Apoptotic Cells Require the Cytohesin GRP-1 in Caenorhabditis elegans. Genetics. 2014;198:229.

3 Denning DP, Hatch V, Horvitz HR. Programmed elimination of cells by caspase-independent cell extrusion in C. elegans. Nature. 2012;488:226-230.

4 Nehme R, Grote P, Tomasi T, Loser S, Holzkamp H, Schnabel R et al. Transcriptional upregulation of both egl-1 BH3-only and ced-3 caspase is required for the death of the male-specific CEM neurons. Cell Death Differ. 2010;17:1266-1276.

5 Consortium CeDM. large-scale screening for targeted knockouts in the Caenorhabditis elegans genome. G3 (Bethesda). 2012;2:1415-1425.

6 Fay D, Bender A. Genetic mapping and manipulation: chapter 4--SNPs: introduction and two-point mapping. WormBook. 2006:1-7.

7 Mello C, Fire A. DNA transformation. Methods Cell Biol. 1995;48:451-482.

8 Dokshin GA, Ghanta KS, Piscopo KM, Mello CC. Robust Genome Editing with Short Single-Stranded and Long, Partially Single-Stranded DNA Donors in Caenorhabditis elegans. Genetics. 2018;210:781-787.

9 Ward JD. Rapid and precise engineering of the Caenorhabditis elegans genome with lethal mutation co-conversion and inactivation of NHEJ repair. Genetics. 2015;199:363-377.

10 Fire A, Xu S, Montgomery MK, Kostas SA, Driver SE, Mello CC. Potent and specific genetic interference by double-stranded RNA in Caenorhabditis elegans. Nature. 1998;391:806-811.

11 Kamath RS, Fraser AG, Dong Y, Poulin G, Durbin R, Gotta M et al. Systematic functional analysis of the Caenorhabditis elegans genome using RNAi. Nature. 2003;421:231-237.

12 Ellis HM, Horvitz HR. Genetic control of programmed cell death in the nematode C. elegans. Cell. 1986;44:817-829.

13 Hoeppner DJ, Hengartner MO, Schnabel R. Engulfment genes cooperate with ced-3 to promote cell death in Caenorhabditis elegans. Nature. 2001;412:202-206.

14 Schnabel R, Hutter H, Moerman D, Schnabel H. Assessing normal embryogenesis in Caenorhabditis elegans using a 4D microscope: variability of development and regional specification. Dev Biol. 1997;184:234-265.

15 Chi C, Ronai D, Than MT, Walker CJ, Sewell AK, Han M. Nucleotide levels regulate germline proliferation through modulating GLP-1/Notch signaling in C. elegans. Genes Dev. 2016;30:307-320.

16 Yuan J, Shaham S, Ledoux S, Ellis HM, Horvitz HR. The C. elegans cell death gene ced-3 encodes a protein similar to mammalian interleukin-1 beta-converting enzyme. Cell. 1993;75:641-652.

17 Lee ES, Xue D. Caspase protocols in Caenorhabditis elegans. Methods Mol Biol. 2014;1133:101-108.

18 Sugimoto T, Mori C, Takanami T, Sasagawa Y, Saito R, Ichiishi E et al. Caenorhabditis elegans par2.1/mtssb-1 is essential for mitochondrial DNA replication and its defect causes comprehensive transcriptional alterations including a hypoxia response. Exp Cell Res. 2008;314:103-114.

19 Wang Y, Zhang Y, Chen L, Liang Q, Yin XM, Miao L et al. Kinetics and specificity of paternal mitochondrial elimination in Caenorhabditis elegans. Nat Commun. 2016;7:12569.
