# Supplementary material for: Uridine 5’-monophosphate (UMP) synthesis connects nucleotide metabolism to programmed cell death in C. elegans
Source: Cell Death Differ. 2025 Sep 3;33(1):25–37. doi: 10.1038/s41418-025-01564-x (PMC12811246; doi:10.1038/s41418-025-01564-x)
Supplement: Supplementary file 3 — Western Blot raw [file 41418_2025_1564_MOESM3_ESM.pdf]

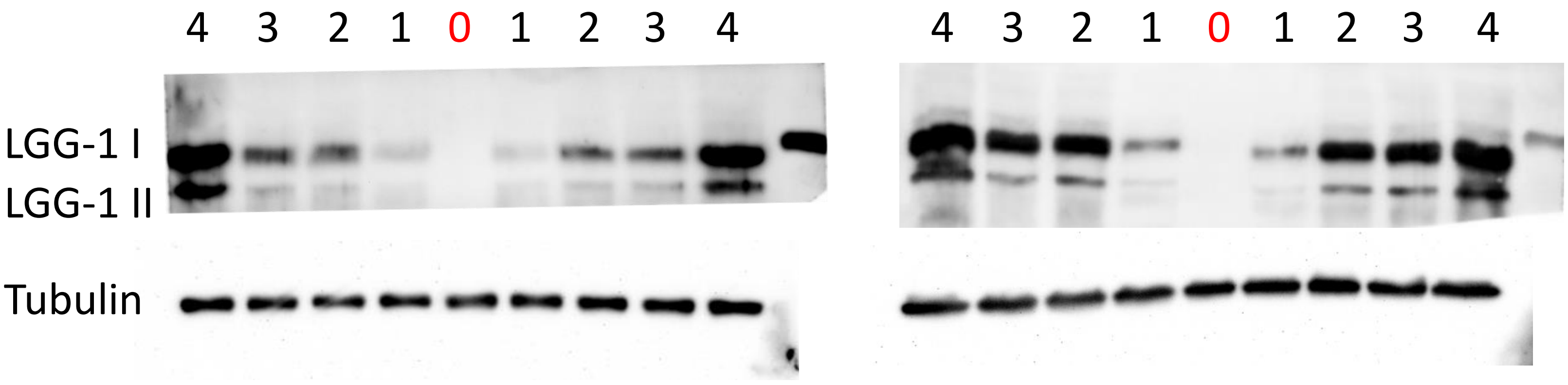

1. *adIs2122[P$_{lgg\text{-}1}$::gfp::lgg-1]*
2. *adIs2122[P$_{lgg\text{-}1}$::gfp::lgg-1]; grp-1*
3. *adIs2122[P$_{lgg\text{-}1}$::gfp::lgg-1]; pyr-1*
4. *adIs2122[P$_{lgg\text{-}1}$::gfp::lgg-1]; pyr-1; grp-1*

0. Wild-type without *adIs2122[P$_{lgg\text{-}1}$::gfp::lgg-1]*

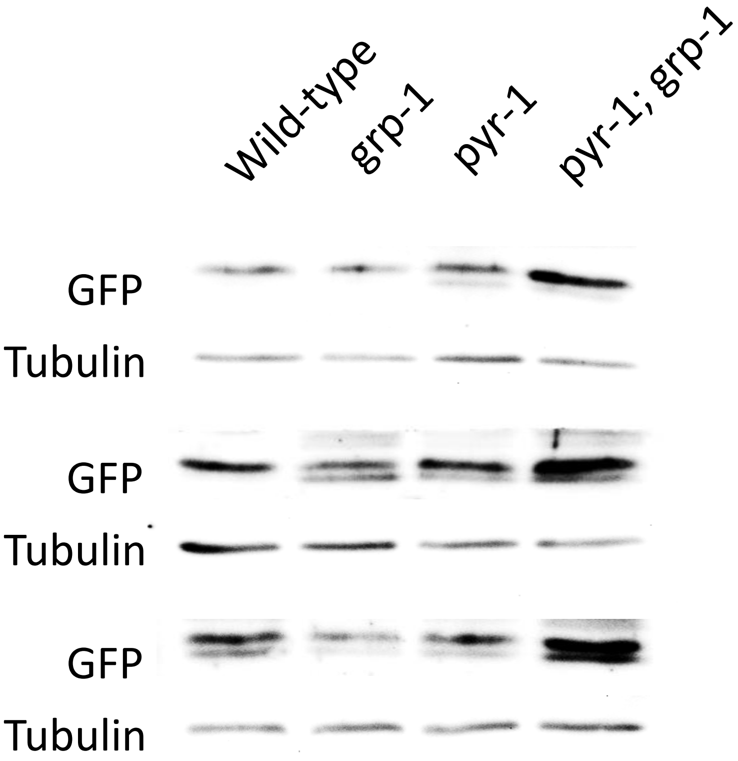
Wild-type
grp-1
pyr-1
pyr-1; grp-1
GFP
Tubulin
GFP
Tubulin
GFP
Tubulin
